# Phenotypic characteristics and transcriptome profile of *Cryptococcus gattii* biofilm

**DOI:** 10.1038/s41598-019-42896-2

**Published:** 2019-04-23

**Authors:** Eliandro Reis Tavares, Bárbara Gionco, Ana Elisa Belotto Morguette, Gabriella Maria Andriani, Alexandre Tadachi Morey, Anderson Oliveira do Carmo, Ulisses de Pádua Pereira, Galdino Andrade, Admilton Gonçalves de Oliveira, Phileno Pinge-Filho, Celso Vataru Nakamura, Lucy Megumi Yamauchi, Sueli Fumie Yamada-Ogatta

**Affiliations:** 1 Programa de Pós-graduação em Microbiologia. Departamento de Microbiologia, Centro de Ciências Biológicas, Universidade Estadual de Londrina, Londrina, Paraná, Brazil; 2 Universidade Norte do Paraná, Londrina, Paraná, Brazil; 3 Instituto Federal do Rio Grande do Sul, Campus Canoas, Canoas, Rio Grande do Sul Brazil; 4 Departamento de Biologia Geral, Instituto de Ciências Biológicas, Universidade Federal de Minas Gerais, Belo Horizonte, Minas Gerais, Brazil; 5 Departamento de Medicina Veterinária Preventiva, Universidade Estadual de Londrina, Londrina, Paraná, Brazil; 6 Departamento de Ciências Patológicas, Centro de Ciências Biológicas, Universidade Estadual de Londrina, Paraná, Brazil; 7 Departamento de Ciências Básicas da Saúde, Centro de Ciências da Saúde, Universidade Estadual de Maringá, Maringá, Paraná, Brazil

**Keywords:** Fungal physiology, Biofilms

## Abstract

In this study, we characterized *Cryptococcus gattii* biofilm formation *in vitro*. There was an increase in the density of metabolically active sessile cells up to 72 h of biofilm formation on polystyrene and glass surfaces. Scanning electron microscopy and confocal laser scanning microscopy analysis revealed that in the early stage of biofilm formation, yeast cells adhered to the abiotic surface as a monolayer. After 12 h, extracellular fibrils were observed projecting from *C. gattii* cells, connecting the yeast cells to each other and to the abiotic surface; mature biofilm consisted of a dense network of cells deeply encased in an extracellular polymeric matrix. These features were also observed in biofilms formed on polyvinyl chloride and silicone catheter surfaces. We used RNA-Seq-based transcriptome analysis to identify changes in gene expression associated with *C. gattii* biofilm at 48 h compared to the free-floating planktonic cells. Differential expression analysis showed that 97 and 224 transcripts were up-regulated and down-regulated in biofilm, respectively. Among the biological processes, the highest enriched term showed that the transcripts were associated with cellular metabolic processes, macromolecule biosynthetic processes and translation.

## Introduction

Microbial biofilms are highly structured communities of surface-attached microorganisms (sessile cells) encased in a self-produced extracellular polymeric matrix (EPM). Biofilms provide microorganisms with numerous advantages, including protection from predators and environmental stresses (such as those caused by variations in pH, temperature and nutrient availability) and enhanced cell-to-cell interactions^1–4^. The biofilm mode of growth is predominantly found in nature and also represents a key strategy in the pathogenesis of various microorganisms in animal and plant hosts^2^. Clinically important, sessile cells of infectious pathogens exhibit reduced sensitivity to antimicrobials and host defense mechanisms^5–14^, leading to persistent and difficult to treat infections^2,13,14^. Crucially, biofilm-related human infections are associated with high mortality rates^15^.

A wide variety of microbial species have been shown to colonize different biotic and abiotic surfaces and form biofilms^1–14^. However, most of our knowledge of fungal biofilms comes from studies of *Candida albicans*^7,12,16–21^ and *Cryptococcus neoformans*^3–5,9–11,22–24^.

*Cryptococcus gattii* is an encapsulated fungus, which can be found in the soil mostly associated with various species of trees^25–27^. Initially, this fungal species was found in tropical and subtropical regions, but the expansion to temperate climate regions has been increasingly reported^28–31^. Remarkably, this species has emerged globally as an important causative agent of cryptococcosis in both immunocompetent hosts and those with a compromised immune system^25,29–32^. Like its sibling species *C. neoformans*, *C. gattii* is equipped with a wide arsenal of virulence determinants, and the main ones include the ability to grow at human body temperature; a polysaccharide capsule (participates in the prevention of phagocytosis by human phagocyte cells and modulation of the immune response); expression of the enzymes laccase (responsible for the production of melanin), superoxide dismutase (provides protection against oxidative burst), phospholipase (participates in the destruction of surfactant molecules) and urease (participates in lung escape and dissemination to the central nervous system); and biofilm formation (infection by sessile cells of *C. gattii* causes a higher mortality rate of *Galleria melonella* larvae, a non-vertebrate model used for fungus-host interaction studies, when compared to infection caused by planktonic cells)^5,33,34^.

Infections caused by *C. gattii* have some similar features as those caused by *C. neoformans* and begin after inhalation of cryptococcal spores from the environment. However, a number of clinical features can distinguish the two infections in humans, where prevalence for a specific organ seems to depend on the geographical region and genotype of the fungus. In general, *C. gattii* infection shows the following characteristics: occurs more often in immunocompetent individuals; presents as an acute infection rather than reactivation disease; involves more often the lung, alone or concurrently with brain infection, where it frequently produces disease with focal biofilm-like structures known as cryptococcomas; produces large-mass lesions associated with central nervous system (CNS) complications, requiring prolonged therapy and/or surgical intervention^29–32^. *C. gattii* also differs in antifungal susceptibility profile, exhibiting higher minimum inhibitory concentrations of azoles, amphotericin B and fluocytosine^35^. Clinical observations have been corroborated by studies in murine models of *C. gattii* inhalation infections; the fungus tends to cause focal lung disease and ultimately fatal pneumonia more frequently than dissemination to the CNS, compared to *C. neoformans*^36^.

Several lines of evidence support the notion that *C. gattii* forms biofilm in both the natural niche^37^ and the mammalian host^22^ as a strategy for survival in different hostile conditions. Indeed, increased incidence of cryptococcomas in the lung and the brain has been observed during infections by *C. gattii*^29–31^. Besides, the use of implanted medical devices for life support therapy, mainly in individuals presenting intracranial hypertension associated with criptococcal meningoencephalitis^29,38^, indicates the importance of investigating the biofilm-forming properties of this microbial species. Currently, the mechanisms employed by *C. gattii* in the formation of the biofilm complex community remain largely unexplored. Therefore, the aim of our study was to investigate the characteristics of *C. gattii* biofilm by using phenotypic and transcriptomic approaches. The understanding of the mechanisms involved in the formation of *C. gattii* biofilm can contribute to the identification of new targets for developing strategies for its control.

## Results

### *C. gattii* forms biofilm on abiotic surfaces

**Figure 1:**
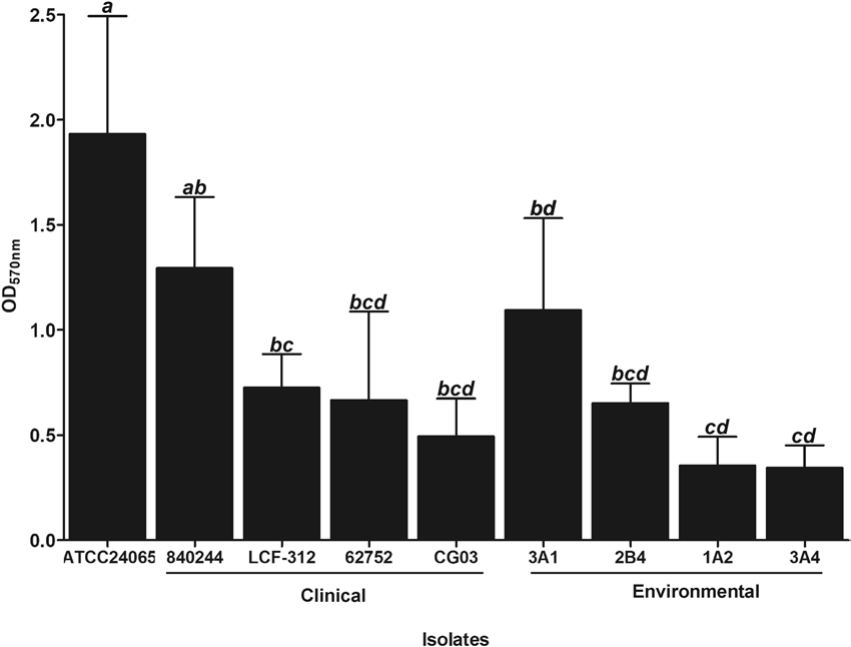
Biofilm formation by clinical (*n* = 4) and environmental (*n* = 4) isolates of *Cryptococcus gattii* complex, and *Cryptococcus gatti* ATCC 24065 reference strain on polystyrene surface incubated in Sabouraud dextrose broth for 72 h at 37 °C. Biofilm biomass was measured after crystal violet staining (OD_570nm_); the values represent the mean ± standard deviation and are representative of three independent experiments. Clinical isolates: 840244, LCF-312, 62752 and CG03; Environmental isolates: 3A1, 2B4, 1A2 and 3A4. Bars not sharing a letter differ significantly (*P* < 0.05) between the isolates.

The ability of the clinical (*n* = 4) and environmental (*n* = 4) isolates of *C. gattii*, and *C. gatti* ATCC 24065 reference strain to form biofilm on polystyrene surface was first analyzed. The biomass of 72-h biofilms was measured after crystal violet staining, and the mean OD_570nm_ (optical density at 570 nm) ± standard deviation was 0.86 ± 0.27, ranging from 0.35 to 1.93. Significant differences (*P* < 0.05) were observed between the different isolates, including those with the same genotype (Fig. 1). Planktonic and sessile cells of the same *Cryptococcus* strain exhibited a clear difference in antifungal susceptibilities. For planktonic cells, the minimum inhibitory concentration (MIC) values of amphotericin B and fluconazole ranged from 0.06 µg/mL to 4.00 µg/mL and 1.00 µg/mL to 64.00 µg/mL, respectively. In contrast, both antifungal agents showed decreased activity against sessile cells, with SMIC_90_ (sessile MIC determined at 90% inhibition) ranging from 16.00 µg/mL to >32.00 µg/mL for amphotericin B, and >128.00 µg/mL for fluconazole for all strains (Table 1). *C. gattii* ATCC 24065 was the highest biofilm-producing strain, so it was chosen for further analyses.

**Table 1:** Antifungal **s**usceptibility profile of clinical (*n* = 4) and environmental (*n* = 4) isolates of *Cryptococcus gattii* complex, and *Cryptococcus gatti* ATCC 24065 reference strain to Amphotericin B and Fluconazole.

Strain	MIC (*µ*g/mL)		SMIC_90_ (*µ*g/mL)	
	AmB	FLZ	AmB	FLZ
*C. gattii* ATCC 24065	0.06	4	32	>128
*C. gattii* 840244	2	32	32	>128
*C.gattii* LCF-312	4	32	32	>128
*C. gattii* 62752	4	16	16	>128
*C. gattii* CG03	4	64	16	>128
*C. gattii* 3A1	0.12	2	32	>128
*C. gattii* 2B4	2	64	32	>128
*C.gattii* 1A2	0.5	1	>32	>128
*C. gattii* 3A4	0.06	16	>32	>128

MIC: Minimal Inhibitory Concentration, as determined by the CLSI (2008) guidelines; SMIC_90_: Sessile Minimal Inhibitory Concentration determined at 90% inhibition compared to antifungal-free control; AmB: Amphotericin B; FLZ: Fluconazole. Clinical isolates: 840244, LCF-312, 62752 and CG03; Environmental isolates: 3A1, 2B4, 1A2 and 3A4.

**Figure 2:**
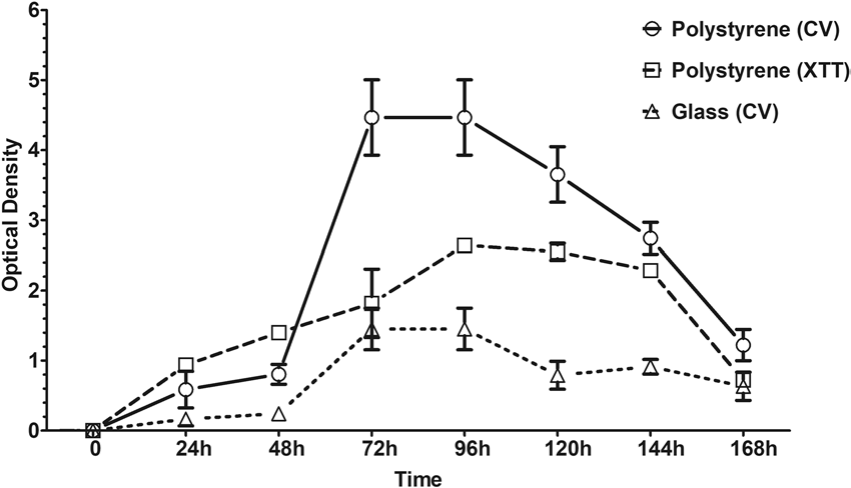
Temporal development of *Cryptococcus gattii* ATCC 24065 biofilm on polystyrene and glass surfaces monitored by measuring the biomass of sessile cells using crystal violet (CV) staining (OD_570nm_) and XTT reduction (OD_492nm_) methods. The values represent the mean ± SD and are representative of three independent experiments.

The temporal development of *C. gattii* ATCC 24065 biofilm was assessed on polystyrene and glass surfaces, and was monitored by measuring the biomass (Fig. 2) of sessile cells using crystal violet staining. *C. gattii* formed greater biofilm on polystyrene surface compared to glass surface (*P* < 0.05). However, the temporal development of the biofilm was similar on both surfaces. In the first 24 h, there was a gradual increase in biomass of sessile cells. After 48 h, a substantial increase in the amount of biomass was observed, reaching a plateau between 72 and 96 h. Afterwards, the amount of biomass gradually decreased. The kinetics of biofilm formation on polystyrene surface was also monitored by measuring the metabolic activity (Fig. 2) of sessile cells using the XTT-reduction assays. Metabolic activity also increased after 24 h, and although it remained high, a plateau was reached after 72 h (Fig. 2). Notably, a drop off of biomass and metabolic activity of sessile cells was observed after 96 h of incubation. In order to verify the effect of nutrient availability on biofilm biomass development, fresh medium was added at 120 and 168 h of incubation. There was a tendency of biofilm biomass increase in the first 24 h; after this period, biofilm biomass decreased again (Fig. S1). These data indicate that nutrient media flow conditions, as can occur in some human body sites, may sustain the sessile cells growth, producing a more “perpetual” biofilm.

**Figure 3:**
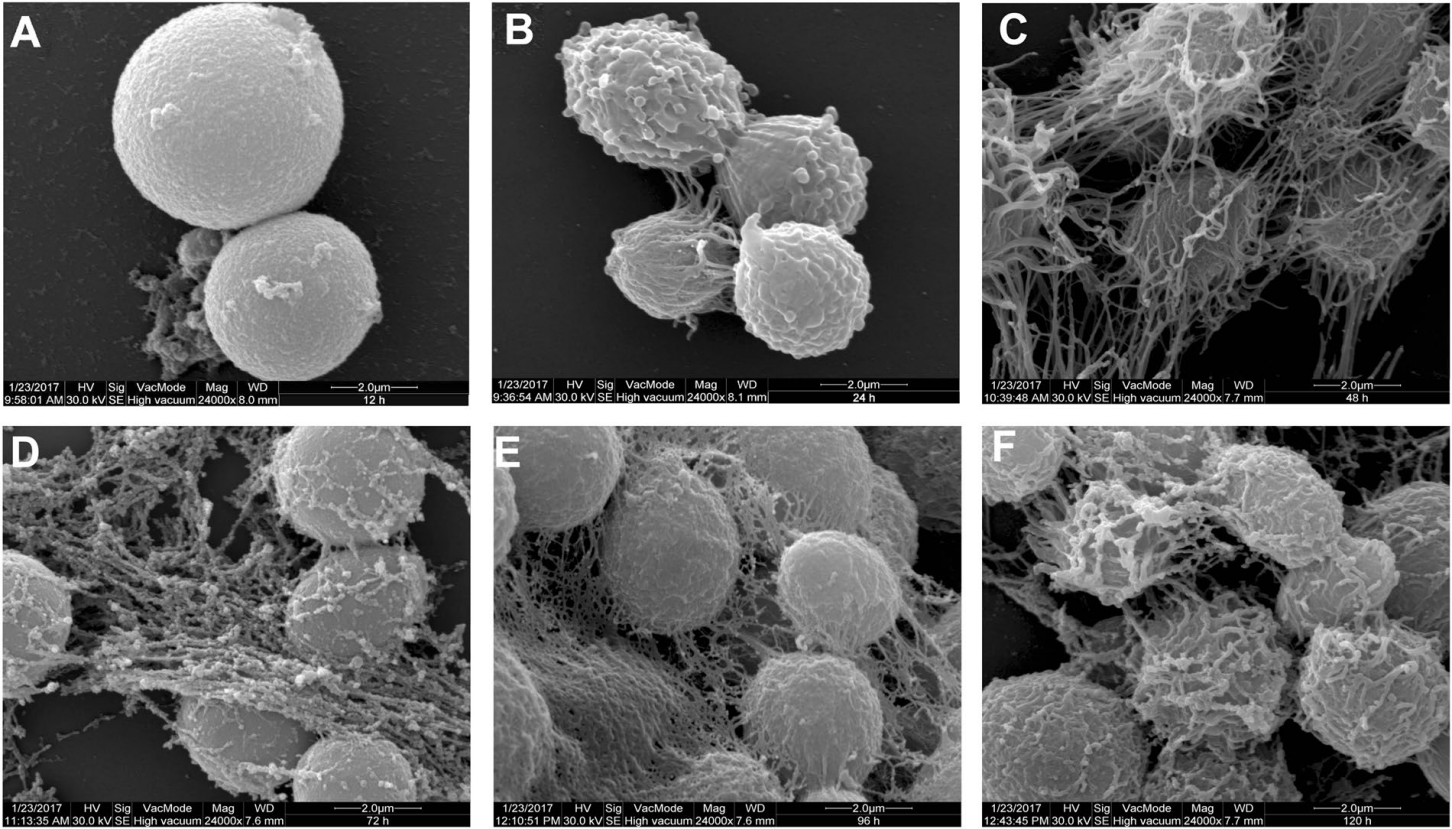
Scanning electron microscopy (SEM) images of *Cryptococcus gattii* ATCC 24065 biofilm formation stages on glass surface over a period of time of incubation at 37 °C. A gradual increase in both, the cell number and the amount of fibrils was observed over time. (**A**) 12 hours; (**B**) 24 hours; (**C**) 48 hours; (**D**) 72 hours; (**E**) 96 hours; and (**F**) 120 hours.

Scanning electron microscopy (SEM) and confocal laser scanning microscopy (CLSM) analyses were used to monitor the morphological and ultrastructural characteristics of *C. gattii* biofilm on glass surface. Early (12–24 h) SEM observations showed yeast cells firmly adhered on the surface as a monolayer. After 12 h, extracellular fibrils were seen projecting from *C. gattii* cells, connecting the yeast cells to each other and to the abiotic surface (Fig. 3A). At 48 to 72 h, both the number of sessile cells and the amount of fibrils surrounding the cells increased significantly with distinct microcolonies forming on the glass surface (Fig. 3C,D), corroborating the data from the crystal violet assay. At 96 to 120 h, the biofilm consisted of a dense network of cells deeply encased in extracellular fibrils that may correspond to the EPM, characterizing the maturation phase (Fig. 3E,F). We also analyzed the ability of *C. gattii* to form biofilm on the silicone (Fig. S2-A) and polyvinyl chloride (Fig. S2-B) catheter surfaces by SEM. As observed in glass, 120 h-biofilms showed yeast cells with extracellular fibrils attached on these surfaces. However, the amount of the fibrils appears to be higher in the biofilm formed on glass as compared to those formed on the catheter surfaces (Fig. S2-ABC).

**Figure 4:**
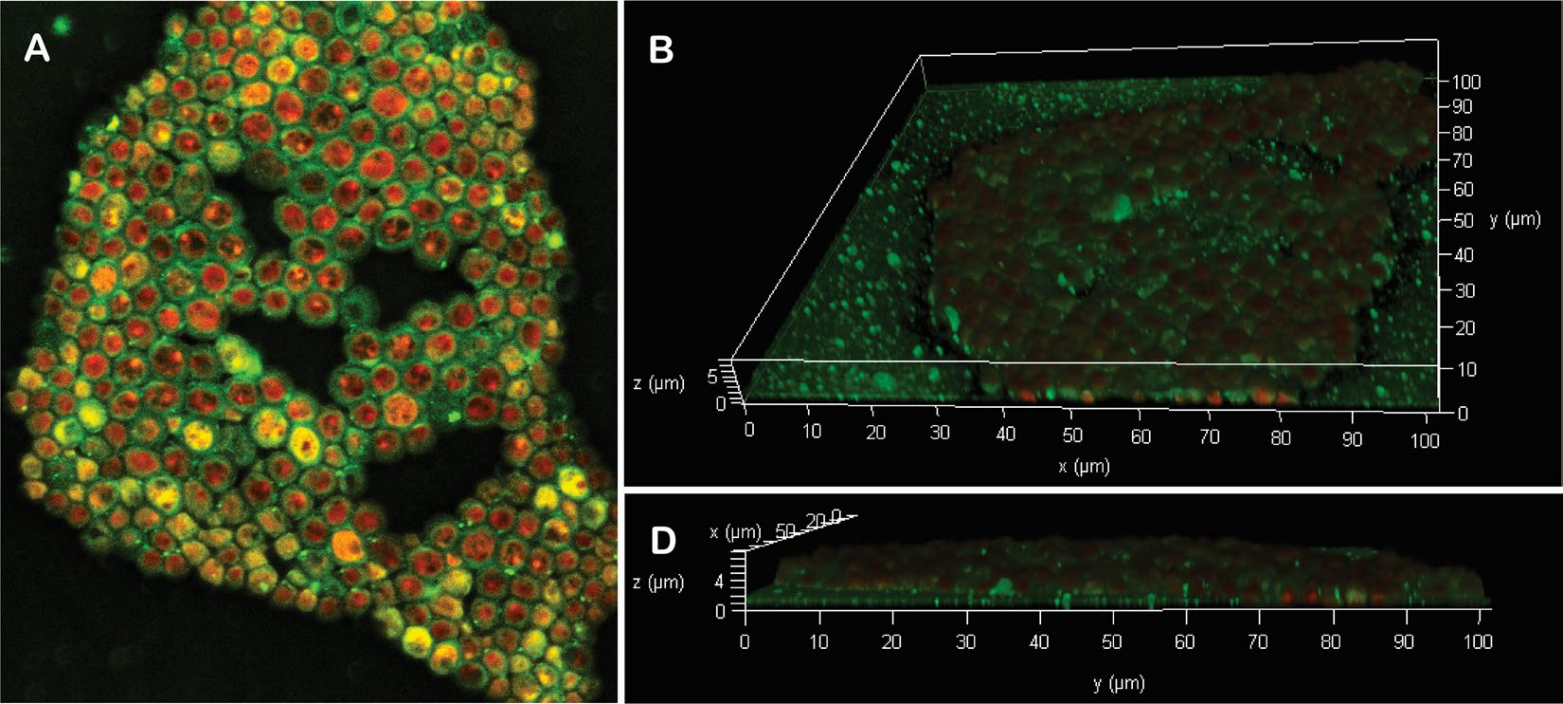
Confocal laser scanning microscopy (CLSM) images of the *Cryptococcus gattii* ATCC 24065 biofilm formed on glass surface after 72 h at 37 °C. Mature biofilm consisted of metabolically active (red-fluorescence due to FUN-1 staining) sessile cells encased in an extracellular polysaccharide-like substance (green-fluorescence due to Con-A staining) organized in an 8-µm-thick monolayer. (**A**) Panoramic view of biofilm; (**B**,**C**) Three-dimensional biofilm reconstitution.

The biofilm of *C. gattii* on glass surface was also observed using CLSM after treatment of the yeast cells with a combination of the fluorescent dyes FUN-1 (a cytoplasmic fluorescent probe that indicates cell viability) and concanavalin A (Con-A, that selectively binds to mannose and glucose residues on the fungal surface) conjugated to Alexa Fluor 488. Figure 4 showed a three-dimensional reconstruction of *C. gattii* 72-h biofilm, resulting from the compilation of a series of individual *xy* sections taken across the *z* axis. The images showed a biofilm organized in an 8-µm-thick monolayer of metabolically active (red-fluorescence due to FUN-1 staining) sessile cells surrounded by an intense green fluorescence (due to Con-A staining), indicating the presence of extracellular polysaccharide-like substance in the EPM.

### Overview of the transcriptome profile of *C. gattii* biofilm formed on polystyrene surface

To gain insights about the transition between planktonic and biofilm lifestyles of *C. gattii*, transcriptome analysis of 48-h biofilm formed on polystyrene surface was performed, and comparisons were made with that from 48-h planktonic counterpart cells. We chose polystyrene, a plastic material that covers a substantial number of surfaces, both for medical applications and in fittings present in hospital settings. A total of 27 million bp (300 bp each) paired-end reads were generated by the Illumina MiSeq^®^ platform. Of these, 14,140,606 and 12,795,000 bp reads corresponded to planktonic and sessile cells, respectively. The fastQC analyses showed Phred Quality Scores (Q) equal to or greater than 30 in 95.0% of total reads representing 99.9% precision and indicating a high quality of the data obtained. Due to the absence of the complete genome of *C. gattii* ATCC 24065, the reads were mapped to the genome of *C. gattii* WM276 (VGI, serotype B, NCBI reference sequence: GCA_000185945.1) and a total of 6,580 transcripts were detected. The mean coverage of the reference genome corresponding to each growth condition was higher than 200-fold, indicating a suitable read number for differential gene expression analyses. Data files were up-loaded to the NCBI Sequence Read Archive (Accession Number: SRP139956).

To detect differentially transcribed genes in 48-h *C. gattii* biofilm, transcriptome data of both growth conditions (48-h biofilm *versus* 48-h planktonic) were analyzed and compared using the RPKM values (fold-change > 1.5 and *P* < 0.001). Overall, 321 transcripts showed significant differences in expression levels in biofilm growth, corresponding to 4.88% of mapped transcripts in this study. Of these, 97 (30.2%) (Table S1) and 224 (69.8%) (Table S2) transcripts were up- and down-regulated in biofilm, respectively. The differences in gene expression in biofilm ranged from 3.63-fold increase (CGB_M3380W, structural constituent of ribosome) up to 72.55-fold increase (CGB_D3260C unknown protein).

### Up-regulated transcripts in biofilm

To identify the cellular pathways affected during 48-h biofilm formation in *C. gattii*, the transcripts were first submitted to Blast2Go software, which consists in searching BLAST hits that are used to map gene ontology (GO). The 97 upregulated transcripts in biofilm were assigned to 136 GO terms, comprising 42 biological processes, 52 molecular functions and 42 cellular components. The GO terms enriched for up-regulated transcripts with known function are shown in Table 2. Among the biological processes, the highest enriched term showed that the transcripts are associated with cellular metabolic processes (mainly protein, peptides and nitrogen compounds), macromolecule biosynthetic process and translation. Individual transcripts are described in Additional Supplementary File (Tables S1 and S2).

**Table 2:** Functional categories of the up-regulated non-ribosomal genes in *Cryptococcus gattii* ATCC 24065 biofilm after 48 h of incubation at 37 °C in Sabouraud broth.

Genes	Fold-Change	BLAST		UNIPROT		Biological process (GO terms)
		Acession	Definition	Acession	Definition	
CGB_A2300C	6,090	ADV19517	MAP kinase phosphatase	E6QZE5	MAP kinase phosphatase	Metabolic process Cellular process Dephosphorylation Phosphorylation Protein dephosphorylation
CGB_I1100W	5,851	ADV24258	Serine-threonine protein kinase IKS1p	E6RBT8	Serine-threonine protein kinase IKS1	Metabolic process Cellular process Phosphorylation
CGB_C6460C	6,931	ADV21181	Aminomethyltransferase,	E6R1S3	Aminomethyltransferase	Metabolic process Cellular process Glycine catabolic process Oxidation-reduction process One-carbon metabolic process Methylation
CGB_E6750W	10,743	ADV22708	Cysteine-type peptidase	E6R6A2	Cysteine-type peptidase	Metabolic process Cellular process Regulation of transcription, DNA-templated Nucleobase-containing compound biosynthetic process Methylglyoxal catabolic process to D-lactate via S-lactoyl-glutathione Lactate biosynthetic process
CGB_I1440W	4,625	ADV24273	Translation initiation factor 5a (eIF-5a)	E6RBX1	Eukaryotic translation initiation factor 5 A	Positive regulation of translational termination Positive regulation of translational elongation Metabolic process Cellular process Translation Translational elongation Translational initiation Translational frameshifting
CGB_A9490W	11,163	ADV24461	Phosphatidylserine decarboxylase	E6QYT2	Phosphatidylserine decarboxylase	Metabolic process Cellular process Phospholipid biosynthetic process
CGB_F1110C	11,326	ADV22883	Phosphatidylserine decarboxylase	E6R7Q0	Phosphatidylserine decarboxylase	Metabolic process Cellular process Phospholipid biosynthetic process
CGB_I0350C	4,195	ADV24237	Squalene monooxygenase	E6RBP8	Squalene monooxygenase	Metabolic process Cellular process Oxidation-reduction process Ergosterol biosynthetic process
CGB_J0030W	4,798	ADV24539	High-affinity glucose transporter of the major facilitator superfamily	E6RCH7	High-affinity glucose transporter of the major facilitator superfamily	Transmembrane transport
CGB_F0090C	14,363	ADV22816	Monocarboxylic acid transporter	E6R7J0	Monocarboxylic acid transporter	Transmembrane transport Ion transmembrane transport
CGB_M2010W	9,461	ADV25506	dUTP diphosphatase	E6RFA0	DUTP diphosphatase	Metabolic process Cellular process Nucleobase-containing compound biosynthetic process dUTP metabolic process
CGB_B1380C	17,155	ADV20191	LSDR Protein	E6R00	LSDR	Metabolic process Oxidation-reduction process
CGB_D0310C	5,072	ADV21505	ABC transporter	E6R5A0	ABC transporter	No GO Terms
CGB_A0280W	6,941	ADV19312	Exonuclease	E6QYY1	Exonuclease	No GO Terms
CGB_A3450C	18,5	ADV19572	DNA repair protein Rad51	E6QXI8	DNA repair protein Rad51	No GO Terms
CGB_D1240C	5,494	ADV21594	Carnitine acetyltransferase	E6R5G7	Carnitine acetyltransferase	No GO Terms
CGB_I0500W	4,06	ADV24217	Oxidoreductase	E6RBR3	Oxidoreductase	No GO Terms
CGB_H2020C	17,777	ADV23943	2,4-dichlorophenoxyacetate alpha-ketoglutarate dioxygenase	E6RAL4	2,4-dichlorophenoxyacetate alpha-ketoglutarate dioxygenase	No GO Terms
CGB_F0420C	7,323	ADV22795	Allergen	E6R7M3	Allergen	No GO Terms
CGB_A9610C	7,007	ADV19988	LEA domain protein	E6QYU4	LEA domain protein	No GO Terms
CGB_C9420C	18,219	ADV21398	Hmp1 protein	E6R2F0	Hmp1 protein	No GO Terms
CGB_B5300W	16	ADV20463	Hydrolase	E6R0X7	D-tyrosyl-tRNA(Tyr) deacylase	No GO Terms
CGB_I0490C	3,934	ADV24231	cytosine deaminase	E6RBR2	Cytosine deaminase	No GO Terms
CGB_M2040C	4,687	ADV25556	E167 tumor protein-like protein	E6RFA3	E167 tumor protein-like protein	No GO Terms

The differentially overexpressed non-ribosomal transcripts in biofilm were searched for homologies in GenBank, using Basic Local Alignment Sequence Tool (BLAST) and UNIPROT databases, separately, to predict molecular functions. Categories and GO terms corresponding to biological process were obtained from analyses with Blast2Go software.

To expand the functional analysis, sequence homology of each upregulated transcript was independently searched in GenBank and Universal Protein Resource (UNIPROT) databases. Of the 97 up-regulated transcripts, 59 (60.8%) matched genes encoding proteins with annotated functions, of which 35 encode ribosomal proteins that participate mainly in ribosome biogenesis and mRNA translation; and 24 encode non-ribosomal proteins (Table 2). The other 38 up-regulated transcripts showed similarity to genes annotated with unknown functions, and these transcripts were up-regulated by more than 3.87-fold up to 72.56-fold (Table 2).

### Validation of transcriptomic analyses

**Figure 5:**
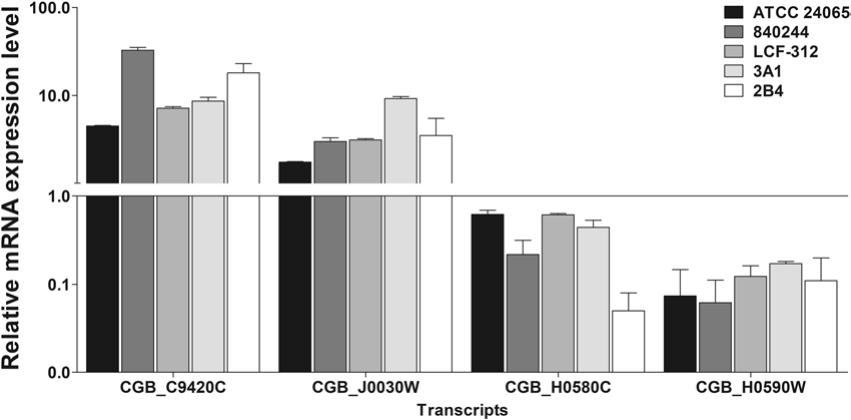
Validation of the data generated by RNA-seq with relative quantitative real-time PCR analysis. mRNAs from planktonic and 48 h-biofilm cells of *Cryptococcus gattii* complex were obtained and the expression of four selected differentially expressed genes in biofilm was quantified by real-time PCR using the QuantiNova SYBR Green RT-PCR system and the cycle threshold method. Changes in transcript levels were determined using *ACT* gene, coding for actin, (Access number: XM_003191370) as an internal control. Results are the mean ± standard error for duplicate determinations and are representative of three independent experiments. Results are presented as the relative gene expression of selected genes related to the control (line). Significant differences were observed between the planktonic and biofilm cells (*P* < 0.05).

To validate the data generated from RNA-seq, two genes that were up-regulated and two that were down-regulated by more than four-fold in biofilm were selected for measuring the changes in expression level of transcripts by quantitative real-time PCR. There was a significant difference (*P* < 0.05) in expression of the selected genes in 48-h biofilm compared to planktonic cells. The expression levels of the genes CGB_C9420C (Hmp1 protein), CGB_J0030W (Hxt4p), CGB_H0580C (Nicotinamide mononucleotide permease) and CGB_H0590W (Tartarate dehydrogenase) are shown in Fig. 5. Although we observed fold differences between the two assays, the changes in gene expression were consistent with those obtained in RNA-seq analysis. Indeed, Person’s correlation coefficient (r) was 0.9946 (*P* < 0.01), indicating a strong correlation between transcript levels detected by qRT-PCR and those revealed by RNA-seq analysis. We also evaluated the expression of selected genes in 48 h-biofilms formed by two clinical (840244 and LCF-312) and environmental (2B4 and 3A1) isolates of *C. gattii* complex on polystyrene surface. As observed in Fig. 5, the pattern of transcript levels of these genes in all isolates was consistent to that of the reference strain, corroborating their differential expression in 48 h-biofilm of this fungal species.

## Discussion

### *C. gattii* forms a highly organized and complex biofilm on abiotic surfaces

Microbial survival in various ecological niches is related to the presence or acquisition of various adaptive attributes, which contribute to withstanding hostile environmental conditions. According to the “ready-made” and “dual use” hypothesis, it has been suggested that human pathogenic fungi possess attributes that play important roles in survival in the natural environment and the other niches^39^. In this context, it is well known that the biofilm mode of growth is universal among all microorganisms in nature, and is a key attribute for survival in different environments^1,2,11,14^.

Indeed, a number of studies have suggested that *C. gattii* adaptation to different growth conditions evolved from selection imposed by the natural ecological niche^37,39^. This fungus is a soil-dwelling organism, and climate such as higher solar radiation and temperature, and environmental predators influence its growth in the environment^39,40^. In this regard, *in vitro* conditions that mimic environmental stress have been shown to be permissive for the biofilm formation by cryptococcal species^3,4^ and adherence of microbial cells to a substratum is the first important step in biofilm development; and several characteristics of both the microbial cell and the substratum can affect this event^2,3,7,23,41,42^.

In this study, we reported the ability of species of the *C. gattii* complex to adhere and form antifungal (amphotericin B and fluconazole) resistant biofilms on polystyrene surface under static conditions, with significant differences in biofilm biomass developed under the same environmental conditions. *C. gattii* ATCC 24065 (VGI, serotype B), which also formed biofilms on glass, silicone and polyvinyl chloride surfaces, produced the largest amount of biomass; therefore, we used this strain to study further characteristics of biofilm development.

Several abiotic materials have been used to study the biofilm formed by different microbial species. Among other parameters, the physicochemical characteristics of the substratum can influence the extent of microorganism attachment and hence biofilm development^2,3,6,7,41^. In our study, glass and polystyrene surfaces supported the biofilm growth of *C. gattii* ATCC 24065. We observed that the biofilm biomass formed on polystyrene by this strain was greater than that developed on glass surface. In contrast, *C. neoformans* B3501, growing in minimal medium, was found to produce increasing amounts of biofilm biomass in the following order: polycarbonate, polystyrene, glass and polyvinyl^3^. The choice of polystyrene and glass surfaces in our study was based on their hydrophobic and hydrophilic properties, respectively; and despite this feature, both surfaces display homogeneous wettability, ensuring microbial adherence^41^; both surface materials are commonly used in *in vitro* assays for evaluating the ability of biofilm formation by different microbial species^3–6,8,12^; these surfaces can be found in different environments, including in healthcare associated setting. Particularly, glass is found in some biomaterials, such as catheters and intracranial pressure monitoring devices that can be used in patients with cryptococcal meningoencephalitis^29,38^.

The formation of biofilms by *C. gattii* ATCC 24065 on polystyrene and glass surfaces proceeded in an organized sequence of events, sharing common steps of development with other biofilm-producing microorganisms, such as: cell adhesion to the surface and formation and maturation of microcolonies^3,5–7,12^. The mature biofilms of *C. gattii*, which had a thickness of 8 µm, consisted of metabolically active yeast cell aggregates highly interwoven in a dense network of extracellular fibrils, possibly constituting the biofilm EPM. Similar morphological features were also observed in biofilms formed on silicone and polyvinyl chloride catheters by this strain. In a previous study, Benaducci *et al*.^5^ described the ability of *C. gattii* ATCC 56990 (VGIII, serotype BC) to form biofilms on polystyrene and glass surfaces. Corroborating our findings, this strain formed biofilms on both surfaces; and 72 h-biofilm formed on glass surface presented a thickness of 5.4 µm, consisting of a dense network of metabolically active cells encased in a self-produced matrix.

Springer *et al*.^37^ reported the formation of extracellular fibrils in *C. gattii* NIH444 (ATCC 32609, serotype B) growing on *Arabidopsis thaliana* leaves, as well as various artificial substrates, including glass coverslips. The formation of these fibrils was associated with both increased virulence of *C. gattii* in nasally infected mice and enhanced fungal resistance to the killing action of human polymorphonuclear neutrophils *in vitro*. Although the nature of these fibrils remains obscure, the authors showed that only encapsulated fungi were able to form these fibrils, and the treatment of these cells with cytoskeleton protein inhibitors resulted in the disorganization of extracellular fibrils. Furthermore, a capsule-deficient *C. gattii* mutant (*cap59∆*) strain lacked extracellular fibrils, impairing both colonization on *A. thaliana* leaves and adhesion to abiotic surface. Finally, these authors suggested that the formation of extracellular fibrils by *C. gattii* is a structural adaptation for cell-to-cell, and/or cell-to-substrate/host interactions.

It has been shown that the capsule is crucial for adhesion of cryptococcal species in biotic and abiotic surfaces^42^. Specifically, the abundant glucuronoxylomannan (GXM) capsular component of *C. neoformans* (serotype D) adhered best, in increasing order to polycarbonate, polystyrene, glass and polyvinyl, correlating with the extent of biofilm biomass on their surfaces^3^. Although we did not analyze the capsular components of *C. gattii* ATCC 24065 (serotype B) in this study, it is known that GXM represents the most abundant capsular component of all *Cryptococcus* species studied so far. Its backbone consists of α1,3 linked mannose residues with xylosyl and glucuronyl side groups, differing in the degree of xylose addition and acetylation in *Cryptococcus* strains. Serotype B capsule shows more xylosyl groups compared to that of serotype D^43^, which may affect the capacity of adhesion on plastic surfaces of cryptococcal strains. Accordingly, Martinez and Casadevall^23^ showed that GXM from serotype D *Cryptococcus* species adhered more strongly to polystyrene compared to serotype A and B capsular types. Moreover, recent report described that genes related to capsule biosynthesis and attachment are differentially expressed between the *C. gattii* lineages (VGI to VGIV)^44^ and this could reflect in their ability to attach and form biofilm on different surfaces.

### *C. gattii* significantly remodels transcriptome profile during biofilm formation on abiotic surface

The transition from a free-living planktonic cell to a sessile, surface-attached mode of growth is a multifactorial process and is driven by a complex and organized cascade of gene expression changes^8,17,21,24^. In fact, biofilm studies of different microbial species report temporal changes in gene expression that can be observed shortly after cell attachment to a substratum^8,21^. Overall, studies reporting the differential gene expression in microbial biofilms have shown that metabolism, transport and stress response are important cellular processes for both the formation and stability of this community^8,18,21,24^.

In this study, the transcriptome analysis of 48-h biofilm of *C. gattii* ATCC 24065 revealed differential expression of genes (up- or down-regulated) involved in various metabolic pathways likely in preparation for the shift in morphology and physiology that follows the biofilm formation. Although there are many types of analyzes that can be performed using the data generated in our study, we discussed only the genes that were up-regulated in the 48-h biofilm. Besides, few studies have focused on gene expression in *C. gattii* so far, thus most of the potential biological functions was inferred from studies carried out with other microbial species.

Interestingly, the gene expression profile of sessile cells growing on the polystyrene surface seemed to mimic that which accounts for survival of the fungus in the environment. Likewise, various biological processes enriched in biofilm in our *in vitro* study were similar to that previously reported in the transcriptome of *C. gattii* R265 recovered from broncoalveolar lavage of infected mice^45^. *C. gattii* can proliferate inside of mouse alveolar macrophages, the first-line defense of host when the fungus arrives in the lung. Thus, antibody-mediated phagocytosis leads to fungal proliferation, agglutination and extrusion from macrophages, (i.e. without any significant damage to the host cells) as biofilm-like microcolonies^22^. These microcolonies can establish in the lung or disseminate to the CNS leading to the formation of cryptococcomas^29–31^. These could represent common pathways involved in biofilm formation by *C. gattii* in distinct niches.

### Up-regulated genes related to information processing and stress response were detected in 48 h-biofilm of *C. gattii*

At 48 h, which corresponds to the growing phase of biofilm of *C. gattii* ATCC 24065, there were a number of up-regulated genes related to information processing (encoding ribosomal proteins and those associated with translation). Ribosomes have a crucial role in supporting cellular growth, and the yeast ribosome content can be tightly adjusted to the synthesis of specific factors involved in energy production and protection against stress under various growth conditions^46^. Particularly, one up-regulated transcript matched the eukaryotic translation initiation factor 5 A (eIF5A), a small acidic protein highly conserved in eukaryotes and essential for cell proliferation. This protein contains the polyamine-derived amino acid hypusine [*N*^ε^-(4-amino-2-hydroxybutyl) lysine], which is formed by a unique post-translational modification. Thus, the activation of eIF5A is strictly dependent on the presence of spermidine^47^. eIF5A enhances the synthesis of proteins containing proline, glycine or charged amino acids^48^; however, its essential function in the translation process is still unclear. It has been shown that this protein plays a direct role in translation of mRNA, stimulating the formation of the first peptide bond^49,50^. However, the contribution of eIF5A in elongation and termination of translation has also been reported^48^. Studies with temperature-sensitive mutants of eIF5A in *Saccharomyces cerevisae* have suggested important roles in cell cycle progression, cell wall integrity, actin depolarization, mRNA decay, protein folding, endoplasmic reticulum-coupled translation and translation of GTPases^47^. Besides the role in these processes, the up-regulation of eIF5A indicates that this protein may regulate the expression of genes involved in biofilm formation in *C. gattii*.

Stress responses are crucial for microbial cells in adapting successfully to a variety of environments. The activation of stress response has been reported in biofilms of *C. albicans*^21^ and *C. neoformans*^24^. We identified one up-regulated gene (CGB_A9610C) encoding the LEA (late embryogenesis abundant) domain protein, a member of the hydrophilin family, that may be involved in the stress response of *C*. *gattii* biofilms. LEA proteins were first described in land plants and are typically induced by exposure to water limitation. LEA-like proteins have also been identified in a number of organisms, including bacteria, most of which inhabit the soil^51^. The function of many LEA proteins is not fully understood, but it seems that these proteins play an important role in cell response to abiotic stresses (particularly dehydration and cold stress) by preventing the inactivation of other cellular proteins and stabilizing membrane structures^52^.

Other up-regulated gene (CGB_A3450C) in *C. gattii* biofilm, which may be involved in response to abiotic stresses, particularly ionizing radiation, encodes the DNA repair protein Rad51. The introduction of DNA breaks (single or double) is one of the lethal effects induced by radiation in living organisms, and to counteract this damage, cells induce the expression of genes involved in DNA repair systems^53^. *Cryptococcus* species have been known to be radiation-tolerant fungi and melanin plays a crucial role in fungus radioprotection due to the combination of its physical shielding and free radical quenching^54^. Along this line, it has been showed that deletion of *RAD51* gene increases *C. neoformans* var. *grubii* H99 susceptibility to both DNA-damaging agents and gamma radiation^55^.

### Up-regulation of genes related to the utilization of alternative carbon sources was observed in *C. gattii* 48 h-biofilm

Combined gene function and biological process analysis of up-regulated genes identified in this study also indicated a shift towards the utilization of alternative carbon sources (other than glucose) such as fatty acids, amino acids, acetate or lactate by *C. gattii* cells during biofilm formation on polystyrene surface. By using a proteomic approach, Santi *et al*.^24^ also identified several up-regulated proteins related to pyruvate metabolism in 48-h sessile cells of *C. neoformans* var. *grubii* H99 strain (serotype A) growing on polystyrene surface. Similarly, up-regulation of genes involved in amino acid, pyruvate and fatty acid metabolic pathways has been previously reported in biofilms of *C. albicans* isolates growing on polymethylmetacrylate and silicone elastomer^21^, and polystyrene^18^ surfaces. The activation of these pathways seems to contribute to the increase in biomass during the stage of biofilm maturation^21^.

Overall, cellular metabolism is fitted to preferentially use nutrients that are assimilated more efficiently or yield more energy. For most organisms, glucose is the preferred carbon source, but when it is absent, the expression of the catabolic pathway for other carbon sources can be switched on^56^. Accordingly, when fungal cells are grown on two-carbon compounds (such as acetate and ethanol) or fatty acids, acetyl-CoA is produced in the cytosol and peroxisomes, respectively, requiring shuttling of acetyl units between intracellular compartments^20^. One up-regulated transcript in *C. gatti* biofilm encodes a putative carnitine acetyltransferase (Cat), an enzyme that mediates the intracellular transport of acetyl-CoA by linking reversibly acetyl units to the carrier molecule *L*-carnitine. Formation of acetyl-carnitine is essential for the transport of acetyl units across the peroxisomal and mitochondrial membranes. In the mitochondria, Cat is required for the conversion of acetyl-carnitine to acetyl-CoA for further metabolism. Deletion of the *CAT2* gene (encoding mitochondrial and peroxisomal carnitine acetyltransferase isoforms) in *C. albicans* resulted in cells unable to grow in the presence of fatty acids, acetate or ethanol. Although this mutant exhibited no growth deficiency on glucose, there was a significant reduction in biofilm biomass formed on polymethylmethacrylate surface for 72 h in the presence of this carbon source^20^. The metabolic shift in 48 h-biofilm of *C. gattii* was also corroborated by the induction of genes involved in protein metabolism (coding for aminomethyltransferase and cysteine-type peptidase). Besides, other genes associated with the maintenance of fungal homeostasis, such as pyrimidine metabolism (coding for cytosine deaminase and dUTP diphosphatase), and biosynthesis of membrane phospholipids (coding for phosphatidylserine decarboxylase) and sterols (coding for squalene monooxygenase) were also up-regulated in *C. gattii* biofilm.

Interestingly, the gene encoding 2,4-dychlorophenoxyacetate alpha-ketoglutarate dioxygenase, the first enzyme of 2,4-dichlorophenoxyacetic acid (2,4 D) catabolism, was highly induced in 48-h biofilm of *C. gattii*. 2,4 D is a synthetic chlorophenoxy compound and is commonly used as broadleaf herbicide in agriculture and forest areas. Several microbial species living in the soil have the ability to catabolize 2,4 D, and the removal of chlorine atoms is a key step in this pathway, which yields intermediates for the tricarboxylic acid cycle^57^. Most of the knowledge about the biodegradation of 2,4 D comes from studies on *Cupriviadus necator*, a Gram-negative soil bacterium, where 2,4-dychlorophenoxyacetate alpha-ketoglutarate dioxygenase (encoded by the *tfdA* gene) catalyzes the removal of the first chlorine atom, yielding 2,4 dichlorophenol (2,4 DCP)^57^. Similarly, the intermediate 2,4 DCP was detected in the culture supernatant of various fungal species. Particularly in *Umbelopsis isabellina*, a soil filamentous fungi, the degradation of 2,4 D is inhibited by the cytochrome P450 inhibitor metyrapone, indicating the involvement of this system in this metabolic pathway^58^.

Consistent with changes in carbon utilization, which demands specific transporters, three up-regulated genes that encode membrane transporters were detected in *C. gattii* biofilms. One transcript encodes a member of the ATP-binding cassette (ABC) and major facilitator superfamily (MFS) membrane-associated transporters. In fungal species, the most studied function of these transporters is related to the efflux of antifungal drugs owing to its clinical significance. Particularly, expression of the genes *AFR1*, *AFR2* and *MDR1*, which encode members of ABC transporters, was up-regulated in *C. gattii* R265 strain treated with fluconazole^59^. Since no antifungal agent was present in the growth medium in our study, we reasoned that this transporter could correlate with fungal metabolism. In fact, ABC/MFS transporters have different substrates and physiological functions that are necessary for the adaptation of the fungi to diverse niches; contributing to the acquisition of nutrients and coping with various stresses, such as heat shock, pH variation, hypoxia, osmotic, oxidative and nitrosative stress, and immune system defenses^60^. Furthermore, a role during biofilm formation has been shown for members of these transporters. Specifically, deletion of the genes *QDR1, QDR2* and *QDR3* (members of MFS) and *CgTPO1_2* (member of MFS) impaired the biofilm architecture and thickness in *Candida albicans*^19^ and *Candida glabrata*^61^, respectively.

The expression of the gene encoding a monocarboxylic acid transporter protein was also up-regulated in *C. gatti* biofilm. Monocarboxylic acids, such as lactate and pyruvate, play an important role in the metabolism of almost all cells of unicellular and multicellular organisms. These molecules can either be used as a carbon source or transported outside cells in response to acid stress conditions^62^. The presence of lactate as carbon source was shown to affect the biofilm-forming capacity of *C. albicans*, increasing its biomass, which was predominantly composed of yeast cells, as compared to glucose-grown biofilms^63^.

### Up-regulated transcript in 48 h-biofilm of *C. gattii* may participate in the cell-to-cell adhesion

The intercellular adhesion is also an important determinant during the development of mature biofilms. Besides the capsule, *C. neoformans* has several attributes that participate in the adhesion process^42^. Wang *et al*.^64^ described the first cryptococcal adhesin, Cfl1 (cell flocculin 1), which plays an important role in filamentation, cell adhesion and virulence of *C. neoformans*. This protein promotes cell-cell aggregation (flocculation), and its secretion is required to function as an adhesin. In this study, one up-regulated transcript matched the Hmp 1 protein, which displayed homology to α-catenin^65^, a protein known to bind to cadherin and play a crucial role in cell-cell adhesion, firmly connecting cells together^66^. Hmp1, along with other proteins related to virulence and response to oxidative stress, were detected in extracellular vesicles produced by *C. neoformans*. It has been suggested that this fungus uses vesicular secretion to deliver virulence factors into the extracellular environment^65^, but Hmp 1 as an adhesin needs to be investigated.

### Conclusion and perspectives

Limitations of this study, which may reduce generalization of the results, are the following: (a) one reference strain was studied, which may not reflect the inherent heterogeneity that exists among *C. gattii* isolates from different sources^67^; (b) data represented the snapshot of the 48-h sessile cell transcriptome and analysis at various stage of biofilm formation can provide the time-dependent changes in genes involved in this process^8,17,21^; (c) the analysis of this study was based on differentially transcribed genes, and changes in mRNA abundance do not always reflect changes in protein content/activity as well as in phenotype^68^; (d) this was an *in vitro* study and the growth medium cannot contain all of the factors found *in vivo*; in fact, several researchers have developed *in vivo* models to characterize and understand the role of microbial biofilms in animal infections^16,69^. In this regard, Andes *et al*.^16^ developed a catheter-associated *C. albicans* biofilm model in rat and reported similar biofilm architecture and antifungal resistance, as observed in those biofilms formed in *in vitro* conditions. However, a faster time course of biofilm development [architectural maturity *in vivo* occurred within the first 24 h *versus* 38–72 h *in vitro*^7,12^]; progressive biofilm growth over the period analyzed (72 h); higher thickness of biofilm [100 µm *in vivo versus* 25–70 µm *in vitro*^7,12^]; and the presence of host cells embedded in EPS were observed in *in vivo* conditions. Moreover, several genes previously identified from *in vitro* biofilm studies were also detected in *in vivo* study^70^. Thus, further studies can reveal if the features reported in our *in vitro* study are also observed in *C. gattii* biofilms formed *in vivo* conditions. Despite these limitations, this is the first study providing an overview of the genes differentially expressed in *C. gattii* VGI serotype B during its transition from the planktonic to 48-h biofilm mode of growth. The annotated transcriptome can be used as a starting point to explore the molecular mechanisms involved in *C. gattii* biofilm formation, extending studies in many directions. In particular, various genes encoding unknown functions were highly expressed in the 48-h biofilm in this study, showing a gap in our knowledge of their role, which may be critically important for biofilm formation by *C. gattii*. Finally, a comprehensive view of the regulatory network involved in biofilm formation can reveal new targets for designing and developing new antifungal agents with antibiofilm activity.

## Material and Methods

### Microorganisms, planktonic growth conditions and antifungal susceptibility test

Reference strain *C. gattii* ATCC 24065 (VGI, serotype B) was kindly donated by Instituto Nacional de Controle de Qualidade – INCQS (FIOCRUZ, Rio de Janeiro, Brazil) and was used in all experiments. Biofilm formation and antifungal susceptibility tests were also evaluated using isolates obtained from the fungal collection of the Laboratório de Biologia Molecular de Microrganismos of Departamento de Microbiologia of Universidade Estadual de Londrina, Paraná, Brazil: four clinical isolates [*C. gattii* CG03 (VGI), *C. gattii* LCF312 (VGI), *C. gattii* 840244 (VGI) and *C. gattii* 62752 (VGII)] and four environmental isolates [*C. gattii* 1A2 (VGI), *C. gattii* 3A1 (VGI), *C. gattii* 3A4 (VGI) and *C. gattii* 2B4 (VGII)]. All fungal strains were grown in Sabouraud dextrose (SD) agar (Himedia, India) at 37 °C for 72 h and stored at 4 °C until processing. Fungi were also stored in SD broth containing 20% glycerol at −80 °C. The isolates were identified by PCR using specific primers complementary to intergenic spacer 2 (IGS2) of ribosomal DNA^26^. Before the experiments, five colony-forming units (CFU) of each microorganism were transferred to SD broth and incubated at 37 °C for 48 h, and the cells were counted with a hemocytometer (Neubauer Improved Chamber). The minimum inhibitory concentrations (MIC) of amphotericin B and fluconazole (Sigma Chemical Co., Brazil) of planktonic cells for reference strain and all isolates were determined by the broth microdilution assay for yeast according to the Clinical and Laboratory Standards Institute guidelines (CLSI)^71^.

### Biofilm formation and antifungal susceptibility of sessile cells

Biofilm formation capacity of all fungi was evaluated in polystyrene, flat-bottomed 96-well microtiter plates (Techno Plastic Products, Switzerland) as described by Martinez and Casadevall^10^, with minor modifications. Briefly, a 20-μL aliquot containing 1.0 × 10^7^ cells was transferred to each well containing 180 μL of SD broth, and the plate was incubated statically at 37 °C for 72 h. Subsequently, the medium was aspirated off and non-adherent cells were removed by washing three times with 0.15 M phosphate-buffered saline (PBS), pH 7.2. Biofilm biomass was dried at room temperature and stained with 0.4% (w/v) crystal violet for 45 min. The stained biofilm was washed with PBS and biofilm-bound stain removed by the addition of 150 µL of 95% methanol (Merck, Brazil). A 100-µL aliquot of bleaching solution was transferred to another plate and the optical density (OD) was measured at 570 nm with a microtiter plate reader (Synergy HT, BioTek, USA)^7^. The OD values of the wells containing no cells (negative controls) were subtracted from the values of test wells to minimize background interference. To determine the antifungal susceptibility of sessile cells, the biofilms of *C. gattii* were formed as described above in RPMI 1640 medium. After 48 h of biofilm formation, the medium was aspirated off and each well was washed three times with sterile PBS. A 200-μL aliquot of medium with 2-fold serial dilutions of antifungal (amphotericin B, 0.06–32.00 μg/mL; fluconazole, 0.125–128.0 μg/mL) was added and the plates were incubated another 48 h at 37 °C. Controls included antifungal-free wells and biofilm-free wells. Sessile MIC was determined at 90% inhibition (SMIC_90_) compared to antifungal-free control wells using the 2,3-bis(2-methoxy-4-nitro-5-sulfo-phenyl)-2H-tetrazolium-5-carboxanilide (XTT)-reduction assay as described elsewhere^9^. A 50-µL aliquot of XTT/menadione solution [1 mg/mL XTT, 1 µM menadione (Sigma-Aldrich, USA)] was added to each well, and the plate was incubated in the dark at 37 °C for 5 h before spectrophotometric readings at 492 nm with a microtiter plate reader. Experiments were carried out in triplicate in three different assays.

### Phenotypic characterization of *C. gattii* biofilm

#### Kinetics of biofilm formation

The kinetics of biofilm formation by *C. gattii* ATCC 24065 was analyzed on polystyrene and glass surfaces. For polystyrene surface, the biofilm was formed as described above, except using flat-bottomed 24-well plates. For glass surface, sterile coverslips of 9 mm in diameter were placed in a 24-well culture plate. A suspension of 1.0 × 10^7^ cells in 1.0 mL of SD broth was added to each well. For both assays, the plates were incubated at 37 °C for various time intervals (24, 48, 72, 96, 120, 144 and 168 h). After each incubation period, biofilm biomass was quantified after staining with 0.4% crystal violet, as described above. For polystyrene surface, the viability of sessile cells was also evaluated using the XTT-reduction assay as described above. The assays were carried out in quintuplicate and performed on three separate occasions.

#### Scanning electron microscopy (SEM)

The biofilm of *C. gattii* ATCC 24065 was formed on glass surface as described above and incubated at 37 °C for various time intervals (12, 24, 48, 72, 96 and 120 h). After each incubation time, the coverslips were recovered and washed gently with PBS. The biofilm was fixed with 2.5% (v/v) glutaraldehyde in 0.1 M sodium cacodylate buffer (pH 7.2) at room temperature. Post-fixation, the cells were dehydrated with a series of ethanol washes (15, 30, 50, 70, 80, 90, 95 and 100%), critical-point dried with CO_2_, coated with gold and examined with a FEI Quanta 200 scanning electron microscope. Strips of polyvinyl chloride and silicone catheters were aseptically cut, placed in wells of 24-well tissue culture plates and processed for biofilm formation as described above. SEM analyses of 120 h-biofilms were performed as described for glass surface.

#### Confocal laser scanning microscopy (CLSM)

The biofilm of *C. gattii* ATCC 24065 was formed on CELLview cell culture dish with glass bottom (Greiner Bio One, Brazil) of 35 mm in diameter, at 37 °C for 72 h. The biofilm growth conditions were as described above for SEM. Afterwards, the biofilm was gently washed once with PBS. Four µL of PBS containing FUN-1 (10 mM, Invitrogen, USA) and concanavalin-A-Alexa Fluor 488 conjugate (ConA; 25 mg/mL, Invitrogen, USA) were placed on the biofilm and the plates incubated for 2 h at room temperature. The biofilm was examined using a confocal laser scanning microscope [objective type: HC PL APO CS2 63x/1.40 oil; magnification: 63x; zoom: 1.03; numerical aperture: 1.40 (Leica Microsystems, Germany)] with the following respective excitation and emission wavelengths: 573 and 592 nm for FUN-1 dye and 498 and 528 nm ConA dye, both with long-pass filter. The intensity used was 17.9 and 14 and the gain was 722 and 714 for FUN-1 and ConA, respectively. FUN-1 is converted from red to orange by metabolically active cells and ConA binds selectively to glucose and mannose residues in the cell wall and matrix^72^.

### Transcriptomic profile of *C. gattii* biofilm

#### Total RNA isolation

For RNA sequencing, total RNA was extracted from biological samples in triplicate (three samples for each growth condition, corresponding to each of three experiments separated spatially and temporally) of planktonic and sessile cells prepared as described below. All samples used for RNA sequencing were grown on 75-cm^2^ polystyrene cell culture flasks (Greiner Bio One, Brazil) containing 40 mL of SD broth. A suspension of 1.0 × 10^7^ cells/mL was added to each flask, and the flasks were incubated at 37 °C. For planktonic cells, the cultures were incubated for 48 h with shaking (200 rpm), and the supernatant was removed by centrifugation at 6,000 *g* for 5 min at 4 °C. For sessile cells, the flasks were incubated for 48 h statically, and adhered cells were harvested by gentle scraping with a sterile rubber policeman, and then centrifuged under the same conditions as above. Both pellets were washed twice with sterile deionized water and an equivalent of 1.0 × 10^8^ cells was used for RNA purification. Total RNA from the cells was purified using the Illustra RNAspin kit (GE Healthcare Life Sciences, United Kingdom) following the manufacturer’s instructions. RNA concentrations were quantified using a spectrophotometer (Synergy HT, BioTek, USA) and RNA quality was determined after 1.5% agarose gel electrophoresis under denaturing conditions^73^.

#### RNA sequencing (RNA-seq)

The construction of cDNA libraries of planktonic and sessile cells was carried out at the Laboratório de Biotecnologia e Marcadores Moleculares of Universidade Federal de Minas Gerais, Belo Horizonte, Minas Gerais, Brazil. Total RNA (1 µg) of each cell growth sample was converted to cDNA using the TruSeq RNA Sample Preparation Kit v.2 (Illumina, USA). The libraries were clustered with TruSeq PE Cluster kit v.2 (Illumina, USA), and quantified by real-time PCR using the KAPA Library Quantification kit Illumina Platforms (KAPA Biosystems, USA). The libraries were normalized to a concentration of 4 nM and sequenced in a MiSeq System (Illumina) with MiSeq Reagent v3 in the paired-end strategy 2 × 300 bp.

#### Reads quality, transcriptome assembly, mapping and annotation

The quality of the reads was evaluated in CLC Genomics Workbench version 8.5.1 platform (CLC bio, Denmark) using the FastQC tool. Due to the high quality of reads, no filter was used to guarantee the whole information about the sequences. Transcriptome assembly, mapping and annotation were also performed in CLC Genomics Workbench with genome-guided strategy. The reads were mapped to the genome of *C. gattii* WM276 (NCBI reference sequence: GCA_000185945.1). Data files were up-loaded to the NCBI Sequence Read Archive (Accession Number: SRP139956).

#### Identification of differentially transcribed genes in biofilm

The data were analyzed by CLC Genomics Workbench platform to quantify expression according to RPKM (Reads per Kilobase of transcript per Million Mapped Reads) values. The differentially transcribed genes between planktonic and sessile cells were then analyzed with Empirical Analysis of the tool DGE^74^. The identified genes were used for normalization and dispersion analyses, and filtered with statistical correction for fold change > 1.5 and *P* < 0.001. The differentially overexpressed non-ribosomal transcripts in biofilm were searched for homologies in GenBank, using Basic Local Alignment Sequence Tool (BLAST) and UNIPROT databases, separately, to predict molecular functions. Results were manually analyzed, and when possible, a functional feature was assigned to the query transcript. In addition, the same transcripts were analyzed by Blast2Go software to determine the general functions, molecular and cellular process.

#### RNA-seq validation by RT-qPCR

To validate the data generated from RNA-seq, two genes each that were up- or down-regulated more than four-fold in biofilm were selected for measuring the changes in expression level of transcripts by quantitative real-time PCR. The nucleotide sequences of selected transcripts were retrieved from GenBank databases, using *C. gattii* WM276 as reference and were as follows: the up-regulated CGB_C9420C and CGB_J0030W that encode the Hmp1 proteins and putative high-affinity glucose transporter of the major facilitator superfamily (Hxt4p), respectively; the down-regulated CGB_H0580C and CGB_H0590W that encode the putative nicotinamide mononucleotide permease and tartarate dehydrogenase proteins, respectively; and *ACT* gene (coding for actin, which was selected for endogenous normalization of expression levels) (Access number: XM_003191370). These sequences were analyzed by BioEdit v.7.2.0 software and used to design specific primers with OligoAnalyzer 3.1 (http://www.idtdna.com) tool. The primer sequences and expected size of amplicons are shown in Table 3. *C. gattii* ATCC 24065, *C. gattii* 840244, *C. gattii* LCF312, *C. gattii* 3A1 and *C. gattii* 2B4 were used in this assay. Total RNA isolation of planktonic and 48 h-biofilm cells were performed as described above. One-step real-time RT-PCR assays were performed in a Rotor-Gene Q 5-Plex apparatus (Qiagen, Germany) using total RNA (200 ng), 1 µM of each primer pair and QuantiNova SYBR Green RT-PCR kit (Qiagen, Brazil), in a final volume of 20 µL according to manufacturer’s recommendations. Cycling conditions were an initial step at 50 °C for 10 min and 95 °C for 2 min, followed by 40 cycles of 95 °C for 30 s, annealing at 60 °C for 30 s and an extension step at 72 °C for 30 s. Thermal dissociation confirmed that RT-PCR generated a single amplicon. After amplification, the Ct (cycle threshold) values were normalized and analyzed on REST2009 software, to determine the expression levels.

**Table 3:** Description of primers used in real-time PCR for quantification of four genes differentially expressed in *Cryptococcus gattii* complex biofilms formed in polystyrene surface.

Transcript	Target	Oligonucleotide	Sequence (5′–3′)
XM_003191370	Actin	Act/Cg-F	GATCTGGCACCATACCTTCTA
		Act/Cg-R	TTCTCTCGGTTCTGCTTGG
CGB_J0030W	High-affinity glucose transporter	Hagt/Cg	CTTCCTTCCCTTCTCACC
		Hagt/Cg	CTTCGGGAGCATCTTCGG
CGB_C9420C	Hmp1 protein	Hmp1/Cg	CAAGGGAGAGGCAGAGATC
		Hmp1/Cg	ACTTGTCACCAGTGATAGCG
CGB_H0590W	Tartarate dehydrogenase	TDH/Cg	AAGTCCAATGCTCAACGAAAT
		TDH/Cg	TCGACCAGCATGTGATCAAG
CGB_H0580C	Nicotinamide mononucleotide permease	MNP/Cg	CAACTCCTTGCTGTTCGTATTT
		MNP/Cg	GGCGAGCTCCTTCTTACTATA

#### Statistical analyses

Statistical analyses were performed using the software GRAPHPAD PRISM, version 6.0 (GRAPHPAD Software, San Diego, CA). Mean OD values of biofilm from all fungi were tested for significance by one-way ANOVA and Dunnett’s multiple comparison *post hoc* test. *P* < 0.05 was considered significant in all cases.

## Supplementary information

Suplementary content

## Data Availability

The data generated and analysed in the current study are included in the article and its Supplementary Information Files. The RNAseq data are publicly available in the National Center for Biotechnology Information (NCBI) Sequence Read Archive under accession SRP139956. Data are also available from the corresponding author on reasonable request.
