# Supplementary material for: Phenotypic characteristics and transcriptome profile of Cryptococcus gattii biofilm
Source: Sci Rep. 2019 Apr 23;9:6438. doi: 10.1038/s41598-019-42896-2 (PMC6478838; doi:10.1038/s41598-019-42896-2)
Supplement: Supplementary file 1 — Suplementary content [file 41598_2019_42896_MOESM1_ESM.pdf]

Phenotypic characteristics and transcriptome profile of *Cryptococcus gattii* biofilm

Eliandro Reis Tavares[1], Bárbara Gionco[2], Ana Elisa Belotto Morguette[1], Gabriella Maria Andriani[1], Alexandre Tadachi Morey[3], Anderson Oliveira do Carmo[4], Ulisses de Pádua Pereira[5], Galdino Andrade[1], Admilton Gonçalves de Oliveira[1]; Phileno Pinge-Filho[6], Celso Vataru Nakamura[7], Lucy Megumi Yamauchi[1], Sueli Fumie Yamada-Ogatta[1,*]

[1]Programa de Pós-graduação em Microbiologia. Departamento de Microbiologia, Centro de Ciências Biológicas, Universidade Estadual de Londrina, Londrina, Paraná, Brazil.

[2]Universidade Norte do Paraná, Londrina, Paraná, Brazil.

[3]Instituto Federal do Rio Grande do Sul, Campus Canoas, Canoas, Rio Grande do Sul, Brazil.

[4]Departamento de Biologia Geral, Instituto de Ciências Biológicas, Universidade Federal de Minas Gerais, Belo Horizonte, Minas Gerais, Brazil.

[5]Departamento de Medicina Veterinária Preventiva, Universidade Estadual de Londrina, Londrina, Paraná, Brazil.

[6]Departamento de Ciências Patológicas, Centro de Ciências Biológicas, Universidade Estadual de Londrina, Paraná, Brazil.

[7]Departamento de Ciências Básicas da Saúde, Centro de Ciências da Saúde, Universidade Estadual de Maringá, Maringá, Paraná, Brazil.

[*]Corresponding author.

Sueli Fumie Yamada-Ogatta. Laboratório de Biologia Molecular de Microrganismos. Departamento de Microbiologia, Centro de Ciências Biológicas, Universidade Estadual de Londrina. Rodovia Celso Garcia Cid, PR445, km380, Campus Universitário. CEP 86057-970. Londrina, Paraná, Brazil. Phone: +55 43 3371-5503. *E*-mail: ogatta@uel.b

Supplementary Content

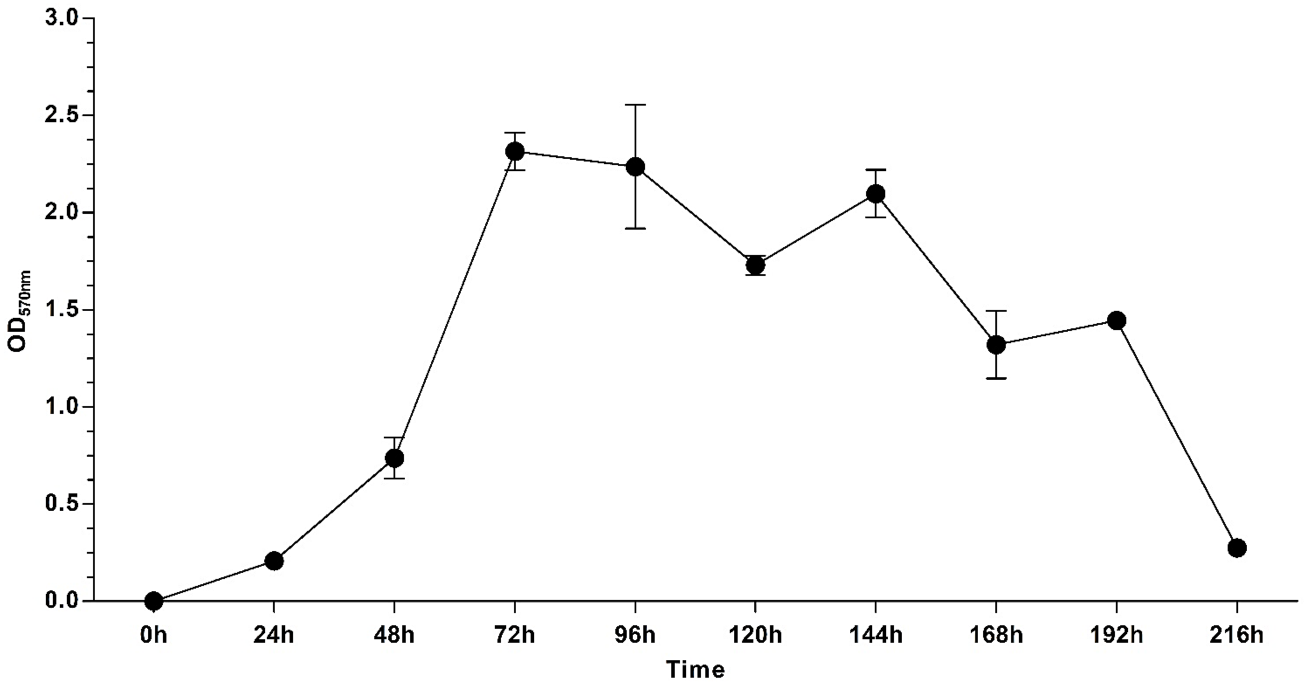

Supplementary Figure S1: Temporal development of *Cryptococcus gattii* ATCC 24065 biofilm on polystyrene surface monitored by measuring the biomass of sessile cells using crystal violet staining (OD_{570nm}) method. The biofilm was formed in flat-bottomed 24-well plates. A suspension of 1.0 x 10^7 cells in 1.0 mL of SD broth was added to each well, and the plates were incubated at 37 ºC for various time intervals (24, 48, 72, 96, 120, 144 and 168 h). Fresh Sabourarud Dextrose medium was added at 120 and 168 h of incubation to evaluate the effect of nutrient availability on biofilm biomass development. After each incubation period, biofilm biomass was quantified after staining with 0.4% crystal violet, as described by Chandra et al., 2001. The values represent the mean ± SD and are representative of three independent experiments performed in quintuplicate.

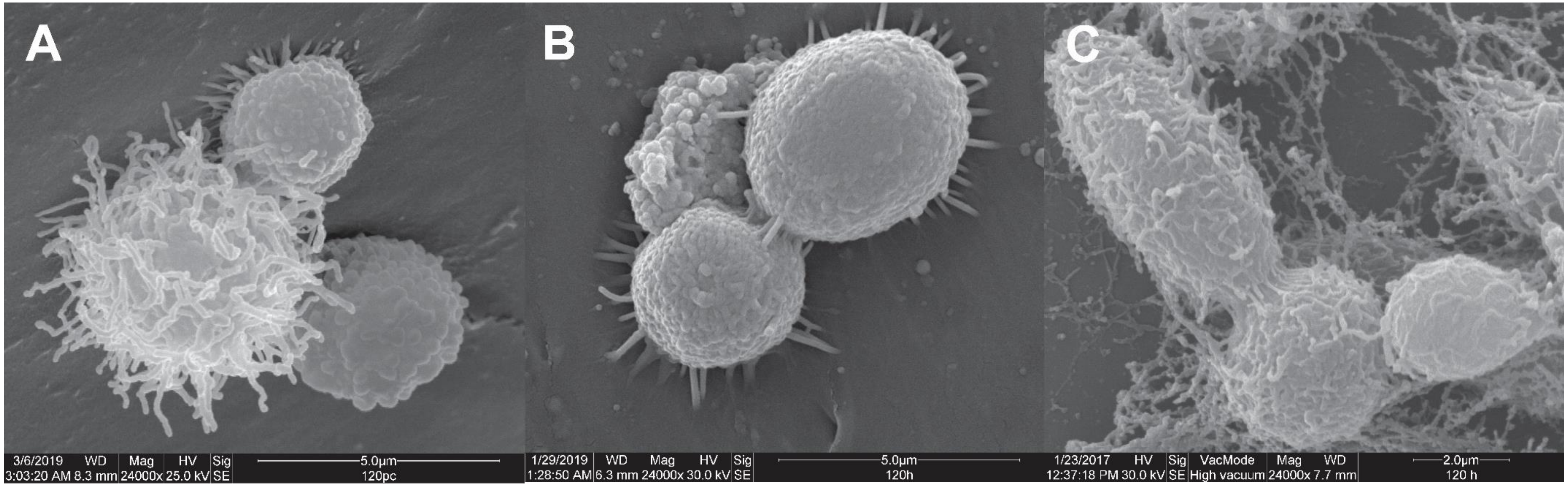

Supplementary Figure S2: Scanning electron microscopy (SEM) images of *Cryptococcus gattii* ATCC 24065 biofilm formation on silicone (A), polyvinyl chloride (B) and glass (C) surfaces over 120 h of incubation at 37 °C. The biofilms were fixed with 2.5% (v/v) glutaraldehyde in 0.1 M sodium cacodylate buffer (pH 7.2) at room temperature. Post-fixation, the cells were dehydrated with a series of ethanol washes (15, 30, 50, 70, 80, 90, 95 and 100%), critical-point dried with CO_2, coated with gold and examined with a FEI Quanta 200 scanning electron microscope.

Supplementary Table S1: Overview of the functional categories of 97 up-regulated transcripts in the *Cryptococcus gattii* ATCC 24065 biofilm after 48 h of incubation at 37 ºC in Sabouraud broth. The differentially overexpressed non-ribosomal transcripts in biofilm were searched for homologies in GenBank, using Basic Local Alignment Sequence Tool (BLAST) and UNIPROT databases, separately, to predict molecular functions. Categories and GO terms corresponding to biological process were obtained from analyses with Blast2Go software.

GO ID	GO term (Biological process)	Sequence Names	Fold-change	BLAST		UniProt	
				Assecion	Definition	Acession	Definition
GO:0045860	Positive regulation of protein kinase activity	CGB_F3630C	6.287366548	ADV22982	Ribosomal protein P2	E6R882	Ribosomal protein P2
		CGB_A7140C	5.42913776	ADV19883	Uncharacterized protein	E6QYA8	Uncharacterized protein
GO:0000028	Ribosomal small subunit assembly	CGB_F4420C	4.816608997	ADV23058	40s ribosomal protein s10	E6R8D3	40s ribosomal protein s10
GO:0042273	Ribosomal large subunit biogenesis	CGB_F4280W	4.656914894	ADV23027	60s ribosomal protein l33-b	E6R8B9	60s ribosomal protein l33-b
		CGB_A0740C	5.889261745	ADV19336	Structural constituent of ribosome	E6QZ27	Structural constituent of ribosome
		CGB_K1570C	3.790697674	ADV24856	Large subunit ribosomal protein	E6RDF2	Large subunit ribosomal protein L3
GO:0045903	Positive regulation of translational fidelity	CGB_E2740C	4.85966736	ADV22436	40S ribosomal protein S9-A	E6R6X2	40S ribosomal protein S9-A
GO:0045905	Positive regulation of translational termination	CGB_I1440W	4.625	ADV24273	Translation initiation factor 5a (eIF-5a)	E6RBX1	Eukaryotic translation initiation factor 5A
GO:0045901	Positive regulation of translational elongation	CGB_I1440W	4.625	ADV24273	Translation initiation factor 5a (eIF-5a)	E6RBX1	Eukaryotic translation initiation factor 5A
GO:0000027	Ribosomal large subunit assembly	CGB_K1570C	3.790697674	ADV24856	Large subunit ribosomal protein	E6RDF2	Large subunit ribosomal protein L3
GO:0008152	Metabolic process	CGB_G2440W	4.77027027	ADV23388	Conserved hypothetical protein	E6R9A8	Uncharacterized protein
		CGB_I0350C	4.195241	ADV24237	Squalene monooxygenase	E6RBP8	Squalene monooxygenase
		CGB_A1030C	5.22133758	ADV19456	60s ribosomal protein l37a	E6QZ34	60s ribosomal protein l37a
		CGB_J0360W	4.612815269	ADV24559	Ribosomal protein S18	E6RCL1	Ribosomal protein S18
		CGB_M2010W	9.461538	ADV25506	dUTP diphosphatase	E6RFA0	DUTP diphosphatase
		CGB_I1100W	5.851613	ADV24258	Serine-threonine protein kinase IKS1p	E6RBT8	Serine-threonine protein kinase IKS1
		CGB_F4280W	4.656914894	ADV23027	60s ribosomal protein l33-b	E6R8B9	60s ribosomal protein l33-b
		CGB_A9370C	5.141600704	ADV19999	60s ribosomal protein l34-b	E6QYS1	60s ribosomal protein l34-b
		CGB_I1190C	5.38372093	ADV24315	RPL39p	E6RBU6	RPL39p
		CGB_A1080C	4.151976936	ADV19453	40s ribosomal protein s3ae-a (s1-a)	E6QZ39	40S ribosomal protein S1

		CGB_G5550W	5.492957746	ADV23596	Conserved hypothetical protein	E6R9Y3	Uncharacterized protein
		CGB_A8680W	5.943830571	ADV20045	60s ribosomal protein l27	E6QYN3	60s ribosomal protein l27
		CGB_K1570C	3.790697674	ADV24856	Large subunit ribosomal protein	E6RDF2	Large subunit ribosomal protein L3
		CGB_B5020W	4.973938224	ADV20449	Structural constituent of ribosome	E6R0U9	Structural constituent of ribosome
		CGB_A6670W	5.157538462	ADV19790	Conserved hypothetical protein	E6QY93	40S ribosomal protein S4
		CGB_I0570W	4.851485149	ADV24223	Conserved hypothetical protein	E6RBS0	Uncharacterized protein
		CGB_A9490W	11.16327	ADV24461	Phosphatidylserine decarboxylase	E6QYT2	Phosphatidylserine decarboxylase
		CGB_E6750W	10.74286	ADV22708	Cysteine-type peptidase	E6R6A2	Cysteine-type peptidase
		CGB_B0250W	4.567672833	ADV20061	60S ribosomal protein l9	E6QZR2	60S ribosomal protein l9
		CGB_A0740C	5.889261745	ADV19336	Structural constituent of ribosome	E6QZ27	Structural constituent of ribosome
		CGB_E2750W	5.019077901	ADV22430	60s ribosomal protein l21-a	E6R6X3	60s ribosomal protein l21-a
		CGB_A7630C	5.029567854	ADV19861	Hypothetical protein CGB_A7630C	E6QYF7	Uncharacterized protein
		CGB_F5590W	5.540013918	ADV23110	40S ribosomal protein S14	E6R8M4	40S ribosomal protein S14
		CGB_K0510C	4.588640275	ADV24780	60S ribosomal protein L23	E6RD65	60S ribosomal protein L23
		CGB_C9370W	4.283018868	ADV21366	Ribosomal protein	E6R2E5	Ribosomal protein
		CGB_D3260C	72.55555556	ADV21741	Hypothetical protein CND01020	E6R5Y4	Uncharacterized protein
		CGB_J0350C	5.85734072	ADV24584	Ribosomal protein L41a	E6RCL0	Ribosomal protein L41a
		CGB_M3380W	3.632496513	ADV25581	Structural constituent of ribosome	E6RFJ1	Structural constituent of ribosome
		CGB_F3630C	6.287366548	ADV22982	Ribosomal protein P2	E6R882	Ribosomal protein P2
		CGB_F1110C	11.32692	ADV22883	Phosphatidylserine decarboxylase	E6R7Q0	Phosphatidylserine decarboxylase
		CGB_B1380C	17.15556	ADV20191	LSDR Protein	E6R00	LSDR
		CGB_H5170W	9.541666667	ADV24110	Hypothetical protein CGB_H5170W	E6RBB7	Uncharacterized protein
		CGB_D3210W	11.96039604	ADV21689	conserved hypothetical protein	E6R5X9	Uncharacterized protein
		CGB_K0690C	4.119651347	ADV24771	Ribosomal protein 22 of the small subunit	E6RD83	Ribosomal protein 22 of the small subunit
		CGB_A7140C	5.42913776	ADV19883	Uncharacterized protein	E6QYA8	Uncharacterized protein

		CGB_M3300W	5.399449036	ADV25577	Protein component of the large (60S) ribosomal subunit	E6RFI3	Protein component of the large (60S) ribosomal subunit
		CGB_E2740C	4.85966736	ADV22436	40S ribosomal protein S9-A	E6R6X2	40S ribosomal protein S9-A
		CGB_C0280W	5.064133017	ADV20725	Structural constituent of ribosome	E6R2K8	Ribosomal protein L15
		CGB_I1440W	4.625	ADV24273	Translation initiation factor 5a (eIF-5a)	E6RBX1	Eukaryotic translation initiation factor 5A
		CGB_C6460C	6.931034	ADV21181	Aminomethyltransferase,	E6R1S3	Aminomethyltransferase
		CGB_C2220C	4.869333333	ADV20920	Ribosomal protein S20	E6R2Z2	Ribosomal protein S20
		CGB_C5630W	6.677192982	ADV21085	40S ribosomal protein S12	E6R1L3	40S ribosomal protein S12
		CGB_A2300C	6.090909	ADV19517	MAP kinase phosphatase	E6QZE5	MAP kinase phosphatase
GO:0009987	Cellular process	CGB_A5330W	5.792592593	ADV19695	Conserved hypothetical protein	E6QXX8	Uncharacterized protein
		CGB_A1030C	5.22133758	ADV19456	60s ribosomal protein l37a	E6QZ34	60s ribosomal protein l37a
		CGB_J0360W	4.612815269	ADV24559	Ribosomal protein S18	E6RCL1	Ribosomal protein S18
		CGB_I0350C	4.195241	ADV24237	Squalene monooxygenase	E6RBP8	Squalene monooxygenase
		CGB_M2010W	9.461538	ADV25506	dUTP diphosphatase	E6RFA0	DUTP diphosphatase
		CGB_I1100W	5.851613	ADV24258	Serine-threonine protein kinase IKS1p	E6RBT8	Serine-threonine protein kinase IKS1
		CGB_F4280W	4.656914894	ADV23027	60s ribosomal protein l33-b	E6R8B9	60s ribosomal protein l33-b
		CGB_A9370C	5.141600704	ADV19999	60s ribosomal protein l34-b	E6QYS1	60s ribosomal protein l34-b
		CGB_I1190C	5.38372093	ADV24315	RPL39p	E6RBU6	RPL39p
		CGB_A1080C	4.151976936	ADV19453	40s ribosomal protein s3ae-a (s1-a)	E6QZ39	40S ribosomal protein S1
		CGB_G5550W	5.492957746	ADV23596	Conserved hypothetical protein	E6R9Y3	Uncharacterized protein
		CGB_A8680W	5.943830571	ADV20045	60s ribosomal protein l27	E6QYN3	60s ribosomal protein l27
		CGB_K1570C	3.790697674	ADV24856	Large subunit ribosomal protein	E6RDF2	Large subunit ribosomal protein L3
		CGB_B5020W	4.973938224	ADV20449	Structural constituent of ribosome	E6R0U9	Structural constituent of ribosome
		CGB_A6670W	5.157538462	ADV19790	Conserved hypothetical protein	E6QY93	40S ribosomal protein S4
		CGB_I0570W	4.851485149	ADV24223	Conserved hypothetical protein	E6RBS0	Uncharacterized protein
		CGB_A9490W	11.16327	ADV24461	Phosphatidylserine decarboxylase	E6QYT2	Phosphatidylserine decarboxylase
		CGB_E6750W	10.74286	ADV22708	Cysteine-type peptidase	E6R6A2	Cysteine-type peptidase

		CGB_B0250W	4.567672833	ADV20061	60S ribosomal protein l9	E6QZR2	60S ribosomal protein l9
		CGB_F4420C	4.816608997	ADV23058	40s ribosomal protein s10	E6R8D3	40s ribosomal protein s10
		CGB_A0740C	5.889261745	ADV19336	structural constituent of ribosome	E6QZ27	Structural constituent of ribosome
		CGB_E2750W	5.019077901	ADV22430	60s ribosomal protein l21-a	E6R6X3	60s ribosomal protein l21-a
		CGB_A7630C	5.029567854	ADV19861	Hypothetical protein CGB_A7630C	E6QYF7	Uncharacterized protein
		CGB_F5590W	5.540013918	ADV23110	40S ribosomal protein S14	E6R8M4	40S ribosomal protein S14
		CGB_K0510C	4.588640275	ADV24780	60S ribosomal protein L23	E6RD65	60S ribosomal protein L23
		CGB_C9370W	4.283018868	ADV21366	Ribosomal protein	E6R2E5	Ribosomal protein
		CGB_D3260C	72.55555556	ADV21741	Hypothetical protein CND01020	E6R5Y4	Uncharacterized protein
		CGB_J0350C	5.85734072	ADV24584	Ribosomal protein L41a	E6RCL0	Ribosomal protein L41a
		CGB_M3380W	3.632496513	ADV25581	Structural constituent of ribosome	E6RFJ1	Structural constituent of ribosome
		CGB_M2040C	4.687793	ADV25556	E167 tumor protein-like protein	E6RFA3	E167 tumor protein-like protein
		CGB_F3630C	6.287366548	ADV22982	Ribosomal protein P2	E6R882	Ribosomal protein P2
		CGB_F1110C	11.32692	ADV22883	Phosphatidylserine decarboxylase	E6R7Q0	Phosphatidylserine decarboxylase
		CGB_H5170W	9.541666667	ADV24110	Hypothetical protein CGB_H5170W	E6RBB7	Uncharacterized protein
		CGB_K0690C	4.119651347	ADV24771	Ribosomal protein 22 of the small subunit	E6RD83	Ribosomal protein 22 of the small subunit
		CGB_A2300C	6.090909	ADV19517	MAP kinase phosphatase	E6QZE5	MAP kinase phosphatase
		CGB_A7140C	5.42913776	ADV19883	Uncharacterized protein	E6QYA8	Uncharacterized protein
		CGB_M3300W	5.399449036	ADV25577	Protein component of the large (60S) ribosomal subunit	E6RFI3	Protein component of the large (60S) ribosomal subunit
		CGB_E2740C	4.85966736	ADV22436	40S ribosomal protein S9-A	E6R6X2	40S ribosomal protein S9-A
		CGB_C0280W	5.064133017	ADV20725	Structural constituent of ribosome	E6R2K8	Ribosomal protein L15
		CGB_I1440W	4.625	ADV24273	Translation initiation factor 5a (eIF-5a)	E6RBX1	Eukaryotic translation initiation factor 5A
		CGB_C2220C	4.869333333	ADV20920	Ribosomal protein S20	E6R2Z2	Ribosomal protein S20
		CGB_C6460C	6.931034	ADV21181	Aminomethyltransferase,	E6R1S3	Aminomethyltransferase
		CGB_C5630W	6.677192982	ADV21085	40S ribosomal protein S12	E6R1L3	40S ribosomal protein S12

GO:0055085	Transmembrane transport	CGB_J0030W	4.798193	ADV24539	High-affinity glucose transporter of the major facilitator superfamily	E6RCH7	High-affinity glucose transporter of the major facilitator superfamily
		CGB_F0090C	14.36364	ADV22816	Monocarboxylic acid transporter	E6R7J0	Monocarboxylic acid transporter
GO:0061817	Endoplasmic reticulum-plasma membrane tethering	CGB_A5330W	5.792592593	ADV19695	Conserved hypothetical protein	E6QXX8	Uncharacterized protein
GO:0042254	Ribosome biogenesis	CGB_J0360W	4.612815269	ADV24559	Ribosomal protein S18	E6RCL1	Ribosomal protein S18
		CGB_F4280W	4.656914894	ADV23027	60s ribosomal protein l33-b	E6R8B9	60s ribosomal protein l33-b
		CGB_A9370C	5.141600704	ADV19999	60s ribosomal protein l34-b	E6QYS1	60s ribosomal protein l34-b
		CGB_F4420C	4.816608997	ADV23058	40s ribosomal protein s10	E6R8D3	40s ribosomal protein s10
		CGB_A0740C	5.889261745	ADV19336	Structural constituent of ribosome	E6QZ27	Structural constituent of ribosome
		CGB_K1570C	3.790697674	ADV24856	Large subunit ribosomal protein	E6RDF2	Large subunit ribosomal protein L3
GO:0034220	Ion transmembrane transport	CGB_F0090C	14.36364	ADV22816	Monocarboxylic acid transporter	E6R7J0	Monocarboxylic acid transporter
GO:0006546	Glycine catabolic process	CGB_C6460C	6.931034	ADV21181	Aminomethyltransferase,	E6R1S3	Aminomethyltransferase
GO:0006355	Regulation of transcription, DNA-templated	CGB_E6750W	10.74286	ADV22708	Cysteine-type peptidase	E6R6A2	Cysteine-type peptidase
GO:0006869	Lipid transport	CGB_A5330W	5.792592593	ADV19695	Conserved hypothetical protein	E6QXX8	Uncharacterized protein
GO:0006412	Translation	CGB_A1030C	5.22133758	ADV19456	60s ribosomal protein l37a	E6QZ34	60s ribosomal protein l37a
		CGB_J0360W	4.612815269	ADV24559	Ribosomal protein S18	E6RCL1	Ribosomal protein S18
		CGB_F4280W	4.656914894	ADV23027	60s ribosomal protein l33-b	E6R8B9	60s ribosomal protein l33-b
		CGB_A9370C	5.141600704	ADV19999	60s ribosomal protein l34-b	E6QYS1	60s ribosomal protein l34-b
		CGB_I1190C	5.38372093	ADV24315	RPL39p	E6RBU6	RPL39p
		CGB_A1080C	4.151976936	ADV19453	40s ribosomal protein s3ae-a (s1-a)	E6QZ39	40S ribosomal protein S1
		CGB_A8680W	5.943830571	ADV20045	60s ribosomal protein l27	E6QYN3	60s ribosomal protein l27
		CGB_K1570C	3.790697674	ADV24856	Large subunit ribosomal protein	E6RDF2	Large subunit ribosomal protein L3
		CGB_B5020W	4.973938224	ADV20449	Structural constituent of ribosome	E6R0U9	Structural constituent of ribosome
		CGB_A6670W	5.157538462	ADV19790	Conserved hypothetical protein	E6QY93	40S ribosomal protein S4
		CGB_I0570W	4.851485149	ADV24223	Conserved hypothetical protein	E6RBS0	Uncharacterized protein
		CGB_B0250W	4.567672833	ADV20061	60S ribosomal protein l9	E6QZR2	60S ribosomal protein l9

		CGB_A0740C	5.889261745	ADV19336	Structural constituent of ribosome	E6QZ27	Structural constituent of ribosome
		CGB_E2750W	5.019077901	ADV22430	60s ribosomal protein l21-a	E6R6X3	60s ribosomal protein l21-a
		CGB_A7630C	5.029567854	ADV19861	Hypothetical protein CGB_A7630C	E6QYF7	Uncharacterized protein
		CGB_F5590W	5.540013918	ADV23110	40S ribosomal protein S14	E6R8M4	40S ribosomal protein S14
		CGB_K0510C	4.588640275	ADV24780	60S ribosomal protein L23	E6RD65	60S ribosomal protein L23
		CGB_C9370W	4.283018868	ADV21366	Ribosomal protein	E6R2E5	Ribosomal protein
		CGB_J0350C	5.85734072	ADV24584	Ribosomal protein L41a	E6RCL0	Ribosomal protein L41a
		CGB_M3380W	3.632496513	ADV25581	Structural constituent of ribosome	E6RFJ1	Structural constituent of ribosome
		CGB_F3630C	6.287366548	ADV22982	Ribosomal protein P2	E6R882	Ribosomal protein P2
		CGB_K0690C	4.119651347	ADV24771	Ribosomal protein 22 of the small subunit	E6RD83	Ribosomal protein 22 of the small subunit
		CGB_A7140C	5.42913776	ADV19883	Uncharacterized protein	E6QYA8	Uncharacterized protein
		CGB_E2740C	4.85966736	ADV22436	40S ribosomal protein S9-A	E6R6X2	40S ribosomal protein S9-A
		CGB_M3300W	5.399449036	ADV25577	Protein component of the large (60S) ribosomal subunit	E6RFI3	Protein component of the large (60S) ribosomal subunit
		CGB_C0280W	5.064133017	ADV20725	Structural constituent of ribosome	E6R2K8	Ribosomal protein L15
		CGB_I1440W	4.625	ADV24273	Translation initiation factor 5a (eIF-5a)	E6RBX1	Eukaryotic translation initiation factor 5A
		CGB_C2220C	4.869333333	ADV20920	Ribosomal protein S20	E6R2Z2	Ribosomal protein S20
		CGB_C5630W	6.677192982	ADV21085	40S ribosomal protein S12	E6R1L3	40S ribosomal protein S12
GO:0016311	Dephosphorylation	CGB_A2300C	6.090909	ADV19517	MAP kinase phosphatase	E6QZE5	MAP kinase phosphatase
		CGB_G5550W	5.492957746	ADV23596	Conserved hypothetical protein	E6R9Y3	Uncharacterized protein
GO:0006470	Protein dephosphorylation	CGB_A2300C	6.090909	ADV19517	MAP kinase phosphatase	E6QZE5	MAP kinase phosphatase
GO:0032147	Activation of protein kinase activity	CGB_F3630C	6.287366548	ADV22982	Ribosomal protein P2	E6R882	Ribosomal protein P2
GO:0034654	Nucleobase-containing compound biosynthetic process	CGB_M2010W	9.461538	ADV25506	dUTP diphosphatase	E6RFA0	DUTP diphosphatase
		CGB_E6750W	10.74286	ADV22708	Cysteine-type peptidase	E6R6A2	Cysteine-type peptidase
		CGB_D3260C	72.55555556	ADV21741	Hypothetical protein CND01020	E6R5Y4	Uncharacterized protein
GO:0055114	Oxidation-reduction process	CGB_I0350C	4.195241	ADV24237	Squalene monooxygenase	E6RBP8	Squalene monooxygenase
		CGB_B1380C	17.15556	ADV20191	LSDR Protein	E6R00	LSDR
		CGB_D3210W	11.96039604	ADV21689	Conserved hypothetical protein	E6R5X9	Uncharacterized protein

		CGB_C6460C	6.931034	ADV21181	Aminomethyltransferase,	E6R1S3	Aminomethyltransferase
GO:0006414	Translational elongation	CGB_F3630C	6.287366548	ADV22982	Ribosomal protein P2	E6R882	Ribosomal protein P2
		CGB_I1440W	4.625	ADV24273	Translation initiation factor 5a (eIF-5a)	E6RBX1	Eukaryotic translation initiation factor 5A
GO:0006413	Translational initiation	CGB_I1440W	4.625	ADV24273	Translation initiation factor 5a (eIF-5a)	E6RBX1	Eukaryotic translation initiation factor 5A
GO:0006048	UDP-N-acetylglucosamine biosynthetic process	CGB_D3260C	72.55555556	ADV21741	Hypothetical protein CND01020	E6R5Y4	Uncharacterized protein
GO:0046080	dUTP metabolic process	CGB_M2010W	9.461538	ADV25506	dUTP diphosphatase	E6RFA0	DUTP diphosphatase
GO:0006730	One-carbon metabolic process	CGB_C6460C	6.931034	ADV21181	Aminomethyltransferase,	E6R1S3	Aminomethyltransferase
GO:0019243	Methylglyoxal catabolic process to D-lactate via S-lactoyl-glutathione	CGB_E6750W	10.74286	ADV22708	Cysteine-type peptidase	E6R6A2	Cysteine-type peptidase
GO:0019249	Lactate biosynthetic process	CGB_E6750W	10.74286	ADV22708	Cysteine-type peptidase	E6R6A2	Cysteine-type peptidase
GO:0032259	Methylation	CGB_C6460C	6.931034	ADV21181	Aminomethyltransferase,	E6R1S3	Aminomethyltransferase
GO:0008654	Phospholipid biosynthetic process	CGB_A9490W	11.16327	ADV24461	Phosphatidylserine decarboxylase	E6QYT2	Phosphatidylserine decarboxylase
		CGB_F1110C	11.32692	ADV22883	Phosphatidylserine decarboxylase	E6R7Q0	Phosphatidylserine decarboxylase
GO:0016310	Phosphorylation	CGB_F3630C	6.287366548	ADV22982	Ribosomal protein P2	E6R882	Ribosomal protein P2
		CGB_I1100W	5.851613	ADV24258	Serine-threonine protein kinase IKS1p	E6RBT8	Serine-threonine protein kinase IKS1
		CGB_A2300C	6.090909	ADV19517	MAP kinase phosphatase	E6QZE5	MAP kinase phosphatase
		CGB_A7140C	5.42913776	ADV19883	Uncharacterized protein	E6QYA8	Uncharacterized protein
GO:0006633	Fatty acid biosynthetic process	CGB_H5170W	9.541666667	ADV24110	Hypothetical protein CGB_H5170W	E6RBB7	Uncharacterized protein
GO:0006696	Ergosterol biosynthetic process	CGB_I0350C	4.195241	ADV24237	Squalene monooxygenase	E6RBP8	Squalene monooxygenase
GO:0002181	Cytoplasmic translation	CGB_I0570W	4.851485149	ADV24223	Conserved hypothetical protein	E6RBS0	Uncharacterized protein
		CGB_F3630C	6.287366548	ADV22982	Ribosomal protein P2	E6R882	Ribosomal protein P2
		CGB_F4280W	4.656914894	ADV23027	60s ribosomal protein l33-b	E6R8B9	60s ribosomal protein l33-b
		CGB_A7140C	5.42913776	ADV19883	Uncharacterized protein	E6QYA8	Uncharacterized protein
		CGB_A1080C	4.151976936	ADV19453	40s ribosomal protein s3ae-a (s1-a)	E6QZ39	40S ribosomal protein S1
		CGB_A0740C	5.889261745	ADV19336	Structural constituent of ribosome	E6QZ27	Structural constituent of ribosome
		CGB_C0280W	5.064133017	ADV20725	Structural constituent of ribosome	E6R2K8	Ribosomal protein L15
		CGB_A7630C	5.029567854	ADV19861	Hypothetical protein CGB_A7630C	E6QYF7	Uncharacterized protein
		CGB_C2220C	4.869333333	ADV20920	Ribosomal protein S20	E6R2Z2	Ribosomal protein S20

		CGB_B5020W	4.973938224	ADV20449	Structural constituent of ribosome	E6R0U9	Structural constituent of ribosome
GO:0006452	Translational frameshifting	CGB_I1440W	4.625	ADV24273	Translation initiation factor 5a (eIF-5a)	E6RBX1	Eukaryotic translation initiation factor 5A
No GO ID	No GO Terms	CGB_C9420C	18.2193	ADV21398	Hmp1 protein	E6R2F0	Hmp1 protein
		CGB_B5300W	16	ADV20463	Hydrolase	E6R0X7	D-tyrosyl-tRNA(Tyr) deacylase
		CGB_F0420C	7.323177	ADV22795	Allergen	E6R7M3	Allergen
		CGB_D1240C	5.494092	ADV21594	Carnitine acetyltransferase	E6R5G7	Carnitine acetyltransferase
		CGB_D0310C	5.07282	ADV21505	ABC transporter	E6R5A0	ABC transporter
		CGB_A0280W	6.941176	ADV19312	Exonuclease	E6QYY1	Exonuclease
		CGB_A3450C	18.5	ADV19572	DNA repair protein Rad51	E6QXI8	DNA repair protein Rad51
		CGB_I0500W	4.066028	ADV24217	Oxidoreductase	E6RBR3	Oxidoreductase
		CGB_H2020C	17.77778	ADV23943	2,4-dichlorophenoxyacetate alpha-ketoglutarate dioxygenase	E6RAL4	2,4-dichlorophenoxyacetate alpha-ketoglutarate dioxygenase
		CGB_A9610C	7.007042	ADV19988	LEA domain protein	E6QYU4	LEA domain protein
		CGB_I0490C	3.934979	ADV24231	cytosine deaminase	E6RBR2	Cytosine deaminase
		CGB_M2040C	4.687793	ADV25556	E167 tumor protein-like protein	E6RFA3	E167 tumor protein-like protein
		CGB_A7150W	4.05172414	ADV19828	60s ribosomal protein l7	E6QYA9	60s ribosomal protein l7
		CGB_A7300W	4.28307022	ADV19834	Ribosomal protein S11	E6QYC4	Ribosomal protein S11
		CGB_A8590C	5.35945946	ADV19926	Ribosomal protein L35	E6QYM4	Ribosomal protein L35
		CGB_B4390C	4.53946054	ADV20428	Small (40S) ribosomal subunit protein	E6R0R8	Small (40S) ribosomal subunit protein
		CGB_C0230C	4.46894689	ADV20768	60S ribosomal protein L12	E6R2K3	60S ribosomal protein L12
		CGB_C7690W	4.41269841	ADV21239	60S ribosomal protein l5-b	E6R224	60S ribosomal protein l5-b
		CGB_C8660C	4.1689038	ADV21316	Ribosomal protein S19	E6R296	Ribosomal protein S19
		CGB_C9050W	5.67358491	ADV21351	Ribosomal protein L37	E6R2B3	Ribosomal protein L37
		CGB_M1220W	5.20324575	ADV25456	Ribosomal protein L13	E6RF56	Ribosomal protein L13
		CGB_A7670C	4.608888889	ADV19858	Uncharacterized protein	E6QYG1	Uncharacterized protein
		CGB_B0440W	15.05555556	ADV20071	Uncharacterized protein	E6QZT2	Uncharacterized protein
		CGB_B2280C	13.94029851	ADV20286	Uncharacterized protein	E6R078	Uncharacterized protein
		CGB_B3460W	16.54615385	ADV20322	Uncharacterized protein	E6R0I8	Uncharacterized protein
		CGB_B4460W	5.3	ADV20405	Uncharacterized protein	E6R0S5	Uncharacterized protein
		CGB_B6360C	5.037634409	ADV20585	Uncharacterized protein	E6R167	Uncharacterized protein
		CGB_C1500W	5.456375839	ADV20814	Uncharacterized protein	E6R2V0	Uncharacterized protein
		CGB_C3550W	13.28571429	ADV20956	Uncharacterized protein	E6R3A3	Uncharacterized protein

		CGB_C5600W	4.246575342	ADV21083	Uncharacterized protein	E6R1L0	Uncharacterized protein
		CGB_C6400C	4.92224622	ADV21185	Uncharacterized protein	E6R1R7	Uncharacterized protein
		CGB_D2480W	6.21630094	ADV21636	Uncharacterized protein	E6R5T7	Uncharacterized protein
		CGB_D3620C	8.507936508	ADV21721	Uncharacterized protein	E6R3R3	Uncharacterized protein
		CGB_D3650W	9.692307692	ADV21709	Uncharacterized protein	E6R3R6	Uncharacterized protein
		CGB_E0080C	15.28333333	ADV22321	Uncharacterized protein	E6R6B0	Uncharacterized protein
		CGB_E5350C	6.173003802	ADV22658	Uncharacterized protein	E6R5Z1	Uncharacterized protein
		CGB_F2305W	34	ADV22919	Uncharacterized protein	E6R7Z4	Uncharacterized protein
		CGB_F2430W	17.76404494	ADV22912	Uncharacterized protein	E6R807	Uncharacterized protein
		CGB_G0510C	10.02281369	ADV23270	Uncharacterized protein	E6R8X7	Uncharacterized protein
		CGB_G0520W	7.581342435	ADV23253	Uncharacterized protein	E6R8X8	Uncharacterized protein
		CGB_H1420C	7.333333333	ADV23852	Uncharacterized protein	E6RAH6	Uncharacterized protein
		CGB_H2360C	7.25	ADV23927	Uncharacterized protein	E6RAP9	Uncharacterized protein
		CGB_H3290C	35.22727273	ADV24010	Uncharacterized protein	E6RAW8	Uncharacterized protein
		CGB_H3730C	5.857142857	ADV23985	Uncharacterized protein	E6RB12	Uncharacterized protein
		CGB_H5490C	9.473684211	ADV24156	Uncharacterized protein	E6RBE9	Uncharacterized protein
		CGB_I0320C	5.306110103	ADV24239	Uncharacterized protein	E6RBP5	Uncharacterized protein
		CGB_I0390C	3.870304748	ADV24236	Uncharacterized protein	E6RBQ2	Uncharacterized protein
		CGB_N1630W	6.796875	ADV25708	Uncharacterized protein	E6RG23	Uncharacterized protein
		CGB_A5460C	3.892682927	ADV19735	Uncharacterized protein	E6QXZ1	Uncharacterized protein

Supplementary Table S2: Overview of the functional categories of 224 down-regulated transcripts in the *Cryptococcus gattii* ATCC 24065 biofilm after 48 hours of incubation at 37 ºC in Sabouraud broth. The differentially undererexpressed transcripts in biofilm were searched for homologies in GenBank, using Basic Local Alignment Sequence Tool (BLAST) and UNIPROT databases, separately, to predict molecular functions. Categories and GO terms corresponding to biological process were obtained from analyses with Blast2Go software.

GO ID	GO Terms (Biological process)	Sequence Names	Fold-change	BLAST		UniProt	
				Acession	Definition	Acession	Definition
GO:0008152	Metabolic process	CGB_I4040W	-5.942	ADV24468	Uncharacterized protein	E6RCE9	3-O-alpha-D-mannopyranosyl-alpha-D-mannopyranose xylosylphosphotransferase
		CGB_A3090W	-13.206	ADV19532	Conserved hypothetical protein	E6QZK1	Uncharacterized protein
		CGB_N3160W	-25.174	ADV25811	Hypothetical Protein	E6RFL5	Uncharacterized protein
		CGB_G1490C	-14.902	ADV23341	Cytoplasm protein	E6R946	Cytoplasm protein
		CGB_A2050W	-4.555	ADV19457	Carbon utilization by utilization of organic compounds-related protein	E6QZC1	Carbon utilization by utilization of organic compounds-related protein
		CGB_A2220W	-6.341	ADV19468	Transcriptional regulatory protein	E6QZD7	Transcriptional regulatory protein
		CGB_N2330W	-6.061	ADV25757	Acetate kinase	E6RG60	Probable acetate kinase
		CGB_A4050W	-4.561	ADV19594	Hypothetical Protein	E6QXL4	Uncharacterized protein
		CGB_I2500W	-3,829	ADV24349	Map kinase kinase kinase mkh1	E6RC52	Map kinase kinase kinase mkh1
		CGB_B2580W	-3,454	ADV20241	Conserved hypothetical protein	E6R0A8	Uncharacterized protein
		CGB_K1610W	-7,213	ADV24837	Hypothetical protein	E6RDF6	Carboxypeptidase
		CGB_J1670W	-6,9	ADV24625	Glutathione transferase	E6RCV4	Glutathione transferase
		CGB_J0120W	-4,960	ADV24544	Hypothetical Protein	E6RCI8	Uncharacterized protein
		CGB_D7200C	-5,346	ADV22061	Ribosomal protein s21	E6R4J4	40S ribosomal protein S21
		CGB_F0220C	-4,028	ADV22806	SNF1A/AMP-activated protein kinase	E6R7K3	Non-specific serine/threonine protein kinase
		CGB_A3390W	-4,735	ADV19549	Acyltransferase	E6QZN1	Acyltransferase
		CGB_K0310W	-5,540	ADV24739	Hypothetical protein	E6RD45	Uncharacterized protein
		CGB_A4040C	-13,322	ADV19675	Conserved hypothetical protein	E6QXL3	Serine/threonine-protein phosphatase
		CGB_E1210C	-4,233	ADV22386	Coproporphyrinogen oxidase	E6R6J6	Coproporphyrinogen oxidase
		CGB_G6520C	-4,785	ADV23682	Casein kinase II beta chain	E6RA48	Casein kinase II subunit beta
		CGB_E0180W	-4,451	ADV22261	Cyclin-dependent protein kinase inhibitor	E6R6C0	Cyclin-dependent protein kinase inhibitor
		CGB_G4250W	-8,119	ADV23507	Hypothetical protein	E6R9M3	Uncharacterized protein
		CGB_J2450W	-6,263	ADV24685	Uncharacterized protein	E6RCZ9	Uncharacterized protein

		CGB_D5580W	-4,059	ADV21863	Nuclear envelope-endoplasmic reticulum network protein	E6R473	Nuclear envelope-endoplasmic reticulum network protein
		CGB_K3610C	-8,225	ADV24994	Inositolphosphorylceramide-B C-26 hydroxylase (IPC-B hydroxylase)	E6RDV9	Inositolphosphorylceramide-B C-26 hydroxylase (IPC-B hydroxylase)
		CGB_D5330W	-4,082	ADV21849	Protein component of the small (40S) ribosomal subunit, putative Rps13p	E6R448	Protein component of the small (40S) ribosomal subunit, putative Rps13p
		CGB_G0120W	-5,141	ADV23235	Ubiquitin-protein ligase	E6R8T7	Ubiquitin-protein ligase
		CGB_A5510W	-4,508	ADV19703	Conserved hypothetical protein	E6QXZ5	Uncharacterized protein
		CGB_C5520C	-4,152	ADV21102	Mitochondrial intermediate peptidase, mitochondrial precursor	E6R3N0	Mitochondrial intermediate peptidase, mitochondrial precursor
		CGB_F6170W	-4,212	ADV23161	Hypothetical Protein	E6R8Q1	Uncharacterized protein
		CGB_N2130W	-7,35	ADV25746	ATP-dependent Clp protease proteolytic subunit	E6RG38	ATP-dependent Clp protease proteolytic subunit
		CGB_E0680C	-9,03	ADV22294	Mannitol-1-phosphate dehydrogenase	E6R6H0	Mannitol-1-phosphate dehydrogenase
		CGB_B9350W	-3,084	ADV20640	Seryl-tRNA synthetase	E6R1G0	Seryl-tRNA synthetase
		CGB_B4240C	-6,8	ADV20436	Serine/threonine-protein kinase	E6R0Q3	Serine/threonine-protein kinase
		CGB_K4420W	-10,828	ADV25046	Phosphoketolase	E6RE14	Phosphoketolase
		CGB_F6340C	-2,977	ADV23200	Nicotinate-nucleotide adenylyltransferase	E6R8R8	Nicotinate-nucleotide adenylyltransferase
		CGB_E0280W	-8,087	ADV22267	D-amino-acid oxidase	E6R6D0	D-amino-acid oxidase
		CGB_H5450C	-4,594	ADV24159	Vacuole protein	E6RBE5	Vacuole protein
		CGB_H1610W	-5,650	ADV23826	Alpha-1,6-mannosyltransferase	E6RAJ5	Alpha-1,6-mannosyltransferase
		CGB_A2040C	-6,668	ADV19527	Isocitrate dehydrogenase (NADP+)	E6QZC0	Isocitrate dehydrogenase [NADP]
		CGB_A0560C	-5,203	ADV19346	Serine/threonine-protein kinase nrc-2	E6QZ09	Serine/threonine-protein kinase nrc-2
		CGB_B9320C	-3,294	ADV20685	Conserved hypothetical protein	E6R1F7	Uncharacterized protein
		CGB_D7530W	-7,929	ADV22011	WD-repeat protein	E6R4M6	WD-repeat protein
		CGB_A3660C	-10,45	ADV20032	RING zinc finger protein	E6QXK9	RING zinc finger protein
		CGB_C4170W	-4,6	ADV21004	Conserved hypothetical protein	E6R3D2	Uncharacterized protein
		CGB_D8370W	-4,324	ADV22090	Stearoyl-CoA 9-desaturase	E6R4U8	Stearoyl-CoA 9-desaturase
		CGB_C7140C	-3,758	ADV21269	Tryptophan 2,3-dioxygenase	E6R1W9	Tryptophan 2,3-dioxygenase
		CGB_D6300C	-4,394	ADV21973	Hypothetical Protein	E6R4D2	Uncharacterized protein
		CGB_A3050C	-4,608	ADV19591	Hypothetical protein	E6QZJ7	Uncharacterized protein

		CGB_I4550W	-5,942	ADV24489	Conserved hypothetical protein	E6RBK9	Uncharacterized protein
		CGB_M0380C	-6,425	ADV25426	Hypothetical Protein	E6RF14	Uncharacterized protein
		CGB_I2050W	-8,222	ADV24326	Adenine phosphoribosyltransferase	E6RC06	Adenine phosphoribosyltransferase
		CGB_E0170C	-9,565	ADV22317	Hypothetical protein	E6R6B9	Uncharacterized protein
		CGB_I3170W	-5,389	ADV24405	Ribosomal protein	E6RC93	Ribosomal protein
		CGB_F1330C	-4,158	ADV22872	Formin binding protein 3	E6R7S2	Formin binding protein 3
		CGB_C1640C	-5,84	ADV20829	Lipase	E6R2W4	Lipase
		CGB_J0420W	-4,476	ADV24563	Conserved hypothetical protein	E6RCL7	Uncharacterized protein
		CGB_G6110W	-7,539	ADV23646	Mannitol-1-phosphate dehydrogenase	E6RA07	Mannitol-1-phosphate dehydrogenase
GO:0050794	Regulation of cellular process	CGB_J2450W	-6,263	ADV24685	Uncharacterized protein	E6RCZ9	Uncharacterized protein
		CGB_N3160W	-25.174	ADV25811	Hypothetical Protein	E6RFL5	Uncharacterized protein
		CGB_D7530W	-7,929	ADV22011	WD-repeat protein	E6R4M6	WD-repeat protein
		CGB_A4040C	-13,322	ADV19675	Conserved hypothetical protein	E6QXL3	Serine/threonine-protein phosphatase
		CGB_L0550C	-20,875	ADV25133	Uncharacterized protein	E6REL5	Uncharacterized protein
		CGB_D6300C	-4,394	ADV21973	Hypothetical Protein	E6R4D2	Uncharacterized protein
		CGB_G6520C	-4,785	ADV23682	Casein kinase II beta chain	E6RA48	Casein kinase II subunit beta
		CGB_A3050C	-4,608	ADV19591	Hypothetical protein	E6QZJ7	Uncharacterized protein
		CGB_A2060W	-5,677	ADV19458	Conserved hypothetical protein	E6QZC2	Uncharacterized protein
		CGB_A2050W	-4.555	ADV19457	Carbon utilization by utilization of organic compounds-related protein	E6QZC1	Carbon utilization by utilization of organic compounds-related protein
		CGB_A2220W	-6.341	ADV19468	Transcriptional regulatory protein	E6QZD7	Transcriptional regulatory protein
		CGB_A2460W	-3,165	ADV19479	RHEB small monomeric GTPase	E6QZG0	RHEB small monomeric GTPase
		CGB_I2500W	-3,829	ADV24349	Map kinase kinase kinase mkh1	E6RC52	Map kinase kinase kinase mkh1
		CGB_H0200C	-6,259	ADV23784	Rho small monomeric GTPase	E6RA76	Rho small monomeric GTPase
		CGB_G4250W	-8,119	ADV23507	Hypothetical protein	E6R9M3	Uncharacterized protein
		CGB_J0120W	-4,960	ADV24544	Hypothetical Protein	E6RCI8	Uncharacterized protein
		CGB_A0560C	-5,203	ADV19346	Serine/threonine-protein kinase nrc-2	E6QZ09	Serine/threonine-protein kinase nrc-2
		CGB_F0220C	-4,028	ADV22806	SNF1A/AMP-activated protein kinase	E6R7K3	Non-specific serine/threonine protein kinase

		CGB_F1100W	-15,365	ADV22825	Rho small monomeric GTPase	E6R7P9	Rho small monomeric GTPase
GO:0044267	Cellular protein metabolic process	CGB_A4040C	-13,322	ADV19675	Conserved hypothetical protein	E6QXL3	Serine/threonine-protein phosphatase
		CGB_K0310W	-5,540	ADV24739	Hypothetical protein	E6RD45	Uncharacterized protein
		CGB_D7530W	-7,929	ADV22011	WD-repeat protein	E6R4M6	WD-repeat protein
		CGB_A3090W	-13.206	ADV19532	Conserved hypothetical protein	E6QZK1	Uncharacterized protein
		CGB_A3660C	-10,45	ADV20032	RING zinc finger protein	E6QXK9	RING zinc finger protein
		CGB_G0120W	-5,141	ADV23235	Ubiquitin-protein ligase	E6R8T7	Ubiquitin-protein ligase
		CGB_C5520C	-4,152	ADV21102	Mitochondrial intermediate peptidase, mitochondrial precursor	E6R3N0	Mitochondrial intermediate peptidase, mitochondrial precursor
		CGB_F6170W	-4,212	ADV23161	Hypothetical Protein	E6R8Q1	Uncharacterized protein
		CGB_G6520C	-4,785	ADV23682	Casein kinase II beta chain	E6RA48	Casein kinase II subunit beta
		CGB_B9350W	-3,084	ADV20640	Seryl-tRNA synthetase	E6R1G0	Seryl-tRNA synthetase
		CGB_B4240C	-6,8	ADV20436	Serine/threonine-protein kinase	E6R0Q3	Serine/threonine-protein kinase
		CGB_I2500W	-3,829	ADV24349	Map kinase kinase kinase mkh1	E6RC52	Map kinase kinase kinase mkh1
		CGB_I3170W	-5,389	ADV24405	Ribosomal protein	E6RC93	Ribosomal protein
		CGB_K1610W	-7,213	ADV24837	Hypothetical protein	E6RDF6	Carboxypeptidase
		CGB_A0560C	-5,203	ADV19346	Serine/threonine-protein kinase nrc-2	E6QZ09	Serine/threonine-protein kinase nrc-2
		CGB_D5330W	-4,082	ADV21849	Protein component of the small (40S) ribosomal subunit, putative Rps13p	E6R448	Protein component of the small (40S) ribosomal subunit, putative Rps13p
		CGB_D7200C	-5,346	ADV22061	Ribosomal protein s21	E6R4J4	40S ribosomal protein S21
		CGB_F0220C	-4,028	ADV22806	SNF1A/AMP-activated protein kinase	E6R7K3	Non-specific serine/threonine protein kinase
GO:0050896	Response to stimulus	CGB_N3160W	-25.174	ADV25811	Hypothetical Protein	E6RFL5	Uncharacterized protein
		CGB_D7530W	-7,929	ADV22011	WD-repeat protein	E6R4M6	WD-repeat protein
		CGB_A4040C	-13,322	ADV19675	Conserved hypothetical protein	E6QXL3	Serine/threonine-protein phosphatase
		CGB_C4170W	-4,6	ADV21004	Conserved hypothetical protein	E6R3D2	Uncharacterized protein
		CGB_L0550C	-20,875	ADV25133	Uncharacterized protein	E6REL5	Uncharacterized protein
		CGB_B4070C	-2,943	ADV20447	Conserved hypothetical protein	E6R0N6	Uncharacterized protein
		CGB_A3050C	-4,608	ADV19591	Hypothetical protein	E6QZJ7	Uncharacterized protein
		CGB_A2060W	-5,677	ADV19458	Conserved hypothetical protein	E6QZC2	Uncharacterized protein

		CGB_A2050W	-4.555	ADV19457	Carbon utilization by utilization of organic compounds-related protein	E6QZC1	Carbon utilization by utilization of organic compounds-related protein
		CGB_A2460W	-3,165	ADV19479	RHEB small monomeric GTPase	E6QZG0	RHEB small monomeric GTPase
		CGB_I2500W	-3,829	ADV24349	Map kinase kinase kinase mkh1	E6RC52	Map kinase kinase kinase mkh1
		CGB_E0540C	-5,570	ADV22302	Glutathione peroxidase	E6R6F6	Glutathione peroxidase
		CGB_H0200C	-6,259	ADV23784	Rho small monomeric GTPase	E6RA76	Rho small monomeric GTPase
		CGB_A0560C	-5,203	ADV19346	Serine/threonine-protein kinase nrc-2	E6QZ09	Serine/threonine-protein kinase nrc-2
		CGB_F0220C	-4,028	ADV22806	SNF1A/AMP-activated protein kinase	E6R7K3	Non-specific serine/threonine protein kinase
		CGB_C4320C	-7,050	ADV21037	Uncharacterized protein	E6R3F0	Uncharacterized protein
GO:0006810	Transport	CGB_F6350C	-12,159	ADV23199	Gamma-aminobutyric acid transporter		Gamma-aminobutyric acid transporter
		CGB_I4210W	-4,331	ADV24475	V-SNARE	E6RCG6	V-SNARE
		CGB_B1090C	-3,715	ADV20206	ITR1	E6QZX9	ITR1
		CGB_C4170W	-4,6	ADV21004	Conserved hypothetical protein	E6R3D2	Uncharacterized protein
		CGB_E2400W	-11,208	ADV22414	Transmembrane transporter Liz1p	E6R6T8	Transmembrane transporter Liz1p
		CGB_C5520C	-4,152	ADV21102	Mitochondrial intermediate peptidase, mitochondrial precursor	E6R3N0	Mitochondrial intermediate peptidase, mitochondrial precursor
		CGB_B5580W	-4,090	ADV20475	Iron ion transporter	E6R106	Iron ion transporter
		CGB_E5610C	-5,345	ADV22646	Phosphatidylinositol transporter	E6R617	Phosphatidylinositol transporter
		CGB_C5400W	-4,310	ADV21071	Peroxisomal membrane protein pex13 (peroxin-13)	E6R3L8	Peroxisomal membrane protein pex13 (peroxin-13)
		CGB_C4320C	-7,050	ADV21037	Uncharacterized protein	E6R3F0	Uncharacterized protein
GO:0055114	Oxidation-reduction process	CGB_B2580W	-3,454	ADV20241	Conserved hypothetical protein	E6R0A8	Uncharacterized protein
		CGB_E0280W	-8,087	ADV22267	D-amino-acid oxidase	E6R6D0	D-amino-acid oxidase
		CGB_K3610C	-8,225	ADV24994	Inositolphosphorylceramide-B C-26 hydroxylase (IPC-B hydroxylase)	E6RDV9	Inositolphosphorylceramide-B C-26 hydroxylase (IPC-B hydroxylase)
		CGB_E1210C	-4,233	ADV22386	Coproporphyrinogen oxidase	E6R6J6	Coproporphyrinogen oxidase
		CGB_D8370W	-4,324	ADV22090	Stearoyl-CoA 9-desaturase	E6R4U8	Stearoyl-CoA 9-desaturase
		CGB_C7140C	-3,758	ADV21269	Tryptophan 2,3-dioxygenase	E6R1W9	Tryptophan 2,3-dioxygenase
		CGB_A2040C	-6,668	ADV19527	Isocitrate dehydrogenase (NADP+)	E6QZC0	Isocitrate dehydrogenase [NADP]

		CGB_I4550W	-5,942	ADV24489	Conserved hypothetical protein	E6RBK9	Uncharacterized protein
		CGB_E0680C	-9,03	ADV22294	Mannitol-1-phosphate dehydrogenase	E6R6H0	Mannitol-1-phosphate dehydrogenase
		CGB_G6110W	-7,539	ADV23646	Mannitol-1-phosphate dehydrogenase	E6RA07	Mannitol-1-phosphate dehydrogenase
GO:0007165	Signal transduction	CGB_I2500W	-3,829	ADV24349	Map kinase kinase kinase mkh1	E6RC52	Map kinase kinase kinase mkh1
		CGB_A4040C	-13,322	ADV19675	Conserved hypothetical protein	E6QXL3	Serine/threonine-protein phosphatase
		CGB_L0550C	-20,875	ADV25133	Uncharacterized protein	E6REL5	Uncharacterized protein
		CGB_A3050C	-4,608	ADV19591	Hypothetical protein	E6QZJ7	Uncharacterized protein
		CGB_A2060W	-5,677	ADV19458	Conserved hypothetical protein	E6QZC2	Uncharacterized protein
		CGB_A0560C	-5,203	ADV19346	Serine/threonine-protein kinase nrc-2	E6QZ09	Serine/threonine-protein kinase nrc-2
		CGB_F0220C	-4,028	ADV22806	SNF1A/AMP-activated protein kinase	E6R7K3	Non-specific serine/threonine protein kinase
		CGB_A2460W	-3,165	ADV19479	RHEB small monomeric GTPase	E6QZG0	RHEB small monomeric GTPase
		CGB_H0200C	-6,259	ADV23784	Rho small monomeric GTPase	E6RA76	Rho small monomeric GTPase
GO:0016310	Phosphorylation	CGB_I2500W	-3,829	ADV24349	Map kinase kinase kinase mkh1	E6RC52	Map kinase kinase kinase mkh1
		CGB_E0180W	-4,451	ADV22261	Cyclin-dependent protein kinase inhibitor	E6R6C0	Cyclin-dependent protein kinase inhibitor
		CGB_G6520C	-4,785	ADV23682	Casein kinase II beta chain	E6RA48	Casein kinase II subunit beta
		CGB_A0560C	-5,203	ADV19346	Serine/threonine-protein kinase nrc-2	E6QZ09	Serine/threonine-protein kinase nrc-2
		CGB_B4240C	-6,8	ADV20436	Serine/threonine-protein kinase	E6R0Q3	Serine/threonine-protein kinase
		CGB_F0220C	-4,028	ADV22806	SNF1A/AMP-activated protein kinase	E6R7K3	Non-specific serine/threonine protein kinase
		CGB_M0380C	-6,425	ADV25426	Hypothetical Protein	E6RF14	Uncharacterized protein
		CGB_N2330W	-6.061	ADV25757	Acetate kinase	E6RG60	Probable acetate kinase
		CGB_E0170C	-9,565	ADV22317	Hypothetical protein	E6R6B9	Uncharacterized protein
GO:0006355	Regulation of transcription, DNA-templated	CGB_N3160W	-25.174	ADV25811	Hypothetical Protein	E6RFL5	Uncharacterized protein
		CGB_J2450W	-6,263	ADV24685	Uncharacterized protein	E6RCZ9	Uncharacterized protein
		CGB_D7530W	-7,929	ADV22011	WD-repeat protein	E6R4M6	WD-repeat protein
		CGB_G4250W	-8,119	ADV23507	Hypothetical protein	E6R9M3	Uncharacterized protein
		CGB_D6300C	-4,394	ADV21973	Hypothetical Protein	E6R4D2	Uncharacterized protein
		CGB_A3050C	-4,608	ADV19591	Hypothetical protein	E6QZJ7	Uncharacterized protein
		CGB_J0120W	-4,960	ADV24544	Hypothetical Protein	E6RCI8	Uncharacterized protein

		CGB_A2220W	-6.341	ADV19468	Transcriptional regulatory protein	E6QZD7	Transcriptional regulatory protein
GO:0006082	Organic acid metabolic process	CGB_B9320C	-3,294	ADV20685	Conserved hypothetical protein	E6R1F7	Uncharacterized protein
		CGB_E0280W	-8,087	ADV22267	D-amino-acid oxidase	E6R6D0	D-amino-acid oxidase
		CGB_K3610C	-8,225	ADV24994	Inositolphosphorylceramide-B C-26 hydroxylase (IPC-B hydroxylase)	E6RDV9	Inositolphosphorylceramide-B C-26 hydroxylase (IPC-B hydroxylase)
		CGB_C7140C	-3,758	ADV21269	Tryptophan 2,3-dioxygenase	E6R1W9	Tryptophan 2,3-dioxygenase
		CGB_A2040C	-6,668	ADV19527	Isocitrate dehydrogenase (NADP+)	E6QZC0	Isocitrate dehydrogenase [NADP]
		CGB_A2050W	-4.555	ADV19457	Carbon utilization by utilization of organic compounds-related protein	E6QZC1	Carbon utilization by utilization of organic compounds-related protein
		CGB_B9350W	-3,084	ADV20640	Seryl-tRNA synthetase	E6R1G0	Seryl-tRNA synthetase
		CGB_N2330W	-6.061	ADV25757	Acetate kinase	E6RG60	Probable acetate kinase
GO:0006508	Proteolysis	CGB_K1610W	-7,213	ADV24837	Hypothetical protein	E6RDF6	Carboxypeptidase
		CGB_D7530W	-7,929	ADV22011	WD-repeat protein	E6R4M6	WD-repeat protein
		CGB_K0310W	-5,540	ADV24739	Hypothetical protein	E6RD45	Uncharacterized protein
		CGB_H5450C	-4,594	ADV24159	Vacuole protein	E6RBE5	Vacuole protein
		CGB_A3660C	-10,45	ADV20032	RING zinc finger protein	E6QXK9	RING zinc finger protein
		CGB_G0120W	-5,141	ADV23235	Ubiquitin-protein ligase	E6R8T7	Ubiquitin-protein ligase
		CGB_C5520C	-4,152	ADV21102	Mitochondrial intermediate peptidase, mitochondrial precursor	E6R3N0	Mitochondrial intermediate peptidase, mitochondrial precursor
		CGB_N2130W	-7,35	ADV25746	ATP-dependent Clp protease proteolytic subunit	E6RG38	ATP-dependent Clp protease proteolytic subunit
GO:0035556	Intracellular signal transduction	CGB_I2500W	-3,829	ADV24349	Map kinase kinase kinase mkh1	E6RC52	Map kinase kinase kinase mkh1
		CGB_H0200C	-6,259	ADV23784	Rho small monomeric GTPase	E6RA76	Rho small monomeric GTPase
		CGB_A4040C	-13,322	ADV19675	Conserved hypothetical protein	E6QXL3	Serine/threonine-protein phosphatase
		CGB_L0550C	-20,875	ADV25133	Uncharacterized protein	E6REL5	Uncharacterized protein
		CGB_A0560C	-5,203	ADV19346	Serine/threonine-protein kinase nrc-2	E6QZ09	Serine/threonine-protein kinase nrc-2
		CGB_F0220C	-4,028	ADV22806	SNF1A/AMP-activated protein kinase	E6R7K3	Non-specific serine/threonine protein kinase
GO:0006629	Lipid metabolic process	CGB_E0180W	-4,451	ADV22261	Cyclin-dependent protein kinase inhibitor	E6R6C0	Cyclin-dependent protein kinase inhibitor
		CGB_B9320C	-3,294	ADV20685	Conserved hypothetical protein	E6R1F7	Uncharacterized protein
		CGB_C1640C	-5,84	ADV20829	Lipase	E6R2W4	Lipase

		CGB_K3610C	-8,225	ADV24994	Inositolphosphorylceramide-B C-26 hydroxylase (IPC-B hydroxylase)	E6RDV9	Inositolphosphorylceramide-B C-26 hydroxylase (IPC-B hydroxylase)
		CGB_D8370W	-4,324	ADV22090	Stearoyl-CoA 9-desaturase	E6R4U8	Stearoyl-CoA 9-desaturase
GO:0051603	Proteolysis involved in cellular protein catabolic process	CGB_K1610W	-7,213	ADV24837	Hypothetical protein	E6RDF6	Carboxypeptidase
		CGB_K0310W	-5,540	ADV24739	Hypothetical protein	E6RD45	Uncharacterized protein
		CGB_A3660C	-10,45	ADV20032	RING zinc finger protein	E6QXK9	RING zinc finger protein
		CGB_G0120W	-5,141	ADV23235	Ubiquitin-protein ligase	E6R8T7	Ubiquitin-protein ligase
GO:0006357	Regulation of transcription by RNA polymerase II	CGB_N3160W	-25.174	ADV25811	Hypothetical Protein	E6RFL5	Uncharacterized protein
		CGB_G4250W	-8,119	ADV23507	Hypothetical protein	E6R9M3	Uncharacterized protein
		CGB_A3050C	-4,608	ADV19591	Hypothetical protein	E6QZJ7	Uncharacterized protein
		CGB_A2220W	-6.341	ADV19468	Transcriptional regulatory protein	E6QZD7	Transcriptional regulatory protein
GO:0006412	Translation	CGB_I3170W	-5,389	ADV24405	Ribosomal protein	E6RC93	Ribosomal protein
		CGB_D5330W	-4,082	ADV21849	Protein component of the small (40S) ribosomal subunit, putative Rps13p	E6R448	Protein component of the small (40S) ribosomal subunit, putative Rps13p
		CGB_B9350W	-3,084	ADV20640	Seryl-tRNA synthetase	E6R1G0	Seryl-tRNA synthetase
		CGB_D7200C	-5,346	ADV22061	Ribosomal protein s21	E6R4J4	40S ribosomal protein S21
GO:0055085	Transmembrane transport	CGB_F6350C	-12,159	ADV23199	Gamma-aminobutyric acid transporter		Gamma-aminobutyric acid transporter
		CGB_B1090C	-3,715	ADV20206	ITR1	E6QZX9	ITR1
		CGB_B5580W	-4,090	ADV20475	Iron ion transporter	E6R106	Iron ion transporter
		CGB_C5400W	-4,310	ADV21071	Peroxisomal membrane protein pex13 (peroxin-13)	E6R3L8	Peroxisomal membrane protein pex13 (peroxin-13)
GO:0006468	Protein phosphorylation	CGB_I2500W	-3,829	ADV24349	Map kinase kinase kinase mkh1	E6RC52	Map kinase kinase kinase mkh1
		CGB_G6520C	-4,785	ADV23682	Casein kinase II beta chain	E6RA48	Casein kinase II subunit beta
		CGB_A0560C	-5,203	ADV19346	Serine/threonine-protein kinase nrc-2	E6QZ09	Serine/threonine-protein kinase nrc-2
		CGB_F0220C	-4,028	ADV22806	SNF1A/AMP-activated protein kinase	E6R7K3	Non-specific serine/threonine protein kinase
GO:0032259	Methylation	CGB_A3090W	-13.206	ADV19532	Conserved hypothetical protein	E6QZK1	Uncharacterized protein
		CGB_G1490C	-14.902	ADV23341	Cytoplasm protein	E6R946	Cytoplasm protein
		CGB_A5510W	-4,508	ADV19703	Conserved hypothetical protein	E6QXZ5	Uncharacterized protein
		CGB_A4050W	-4,561	ADV19594	Hypothetical Protein	E6QXL4	Uncharacterized protein
GO:0005975	Carbohydrate metabolic process	CGB_I4040W	-5.942	ADV24468	Uncharacterized protein	E6RCE9	3-O-alpha-D-mannopyranosyl-alpha-D-mannopyranose xylosylphosphotransferase

		CGB_J0420W	-4,476	ADV24563	Conserved hypothetical protein	E6RCL7	Uncharacterized protein
		CGB_A2050W	-4.555	ADV19457	Carbon utilization by utilization of organic compounds-related protein	E6QZC1	Carbon utilization by utilization of organic compounds-related protein
		CGB_K4420W	-10,828	ADV25046	Phosphoketolase	E6RE14	Phosphoketolase
GO:0016192	Vesicle-mediated transport	CGB_I4210W	-4,331	ADV24475	V-SNARE	E6RCG6	V-SNARE
		CGB_C4170W	-4,6	ADV21004	Conserved hypothetical protein	E6R3D2	Uncharacterized protein
		CGB_C4320C	-7,050	ADV21037	Uncharacterized protein	E6R3F0	Uncharacterized protein
GO:0016311	Dephosphorylation	CGB_D5580W	-4,059	ADV21863	Nuclear envelope-endoplasmic reticulum network protein	E6R473	Nuclear envelope-endoplasmic reticulum network protein
		CGB_A4040C	-13,322	ADV19675	Conserved hypothetical protein	E6QXL3	Serine/threonine-protein phosphatase
GO:0006633	Fatty acid biosynthetic process	CGB_B9320C	-3,294	ADV20685	Conserved hypothetical protein	E6R1F7	Uncharacterized protein
		CGB_K3610C	-8,225	ADV24994	Inositolphosphorylceramide-B C-26 hydroxylase (IPC-B hydroxylase)	E6RDV9	Inositolphosphorylceramide-B C-26 hydroxylase (IPC-B hydroxylase)
GO:0006116	NADH oxidation	CGB_E0680C	-9,03	ADV22294	Mannitol-1-phosphate dehydrogenase	E6R6H0	Mannitol-1-phosphate dehydrogenase
		CGB_G6110W	-7,539	ADV23646	Mannitol-1-phosphate dehydrogenase	E6RA07	Mannitol-1-phosphate dehydrogenase
GO:0045944	Positive regulation of transcription by RNA polymerase II	CGB_N3160W	-25.174	ADV25811	Hypothetical Protein	E6RFL5	Uncharacterized protein
		CGB_G4250W	-8,119	ADV23507	Hypothetical protein	E6R9M3	Uncharacterized protein
GO:0000462	Maturation of SSU-rRNA from tricistronic rRNA transcript (SSU-rRNA, 5.8S rRNA, LSU-rRNA)	CGB_D5330W	-4,082	ADV21849	Protein component of the small (40S) ribosomal subunit, putative Rps13p	E6R448	Protein component of the small (40S) ribosomal subunit, putative Rps13p
		CGB_D7200C	-5,346	ADV22061	Ribosomal protein s21	E6R4J4	40S ribosomal protein S21
GO:0006067	Ethanol metabolic process	CGB_E0680C	-9,03	ADV22294	Mannitol-1-phosphate dehydrogenase	E6R6H0	Mannitol-1-phosphate dehydrogenase
		CGB_G6110W	-7,539	ADV23646	Mannitol-1-phosphate dehydrogenase	E6RA07	Mannitol-1-phosphate dehydrogenase
GO:0007039	Protein catabolic process in the vacuole	CGB_K1610W	-7,213	ADV24837	Hypothetical protein	E6RDF6	Carboxypeptidase
		CGB_K0310W	-5,540	ADV24739	Hypothetical protein	E6RD45	Uncharacterized protein
GO:0046938	Phytochelatin biosynthetic process	CGB_K1610W	-7,213	ADV24837	Hypothetical protein	E6RDF6	Carboxypeptidase
		CGB_K0310W	-5,540	ADV24739	Hypothetical protein	E6RD45	Uncharacterized protein
GO:0000278	Mitotic cell cycle	CGB_I2500W	-3,829	ADV24349	Map kinase kinase kinase mkh1	E6RC52	Map kinase kinase kinase mkh1
		CGB_F0220C	-4,028	ADV22806	SNF1A/AMP-activated protein kinase	E6R7K3	Non-specific serine/threonine protein kinase
GO:0045859	Regulation of protein kinase activity	CGB_I2500W	-3,829	ADV24349	Map kinase kinase kinase mkh1	E6RC52	Map kinase kinase kinase mkh1

		CGB_G6520C	-4,785	ADV23682	Casein kinase II beta chain	E6RA48	Casein kinase II subunit beta
GO:0033617	Mitochondrial respiratory chain complex IV assembly	CGB_B9020W	-2,856	ADV20624	Hypothetical protein	E6R1C7	Uncharacterized protein
		CGB_A4480W	-8,360	ADV19615	Mitochondrion protein	E6QXQ7	Mitochondrion protein
GO:0006511	Ubiquitin-dependent protein catabolic process	CGB_A3660C	-10,45	ADV20032	RING zinc finger protein	E6QXK9	RING zinc finger protein
		CGB_G0120W	-5,141	ADV23235	Ubiquitin-protein ligase	E6R8T7	Ubiquitin-protein ligase
GO:0006979	Response to oxidative stress	CGB_B4070C	-2,943	ADV20447	Conserved hypothetical protein	E6R0N6	Uncharacterized protein
		CGB_A2050W	-4.555	ADV19457	Carbon utilization by utilization of organic compounds-related protein	E6QZC1	Carbon utilization by utilization of organic compounds-related protein
GO:0042594	Response to starvation	CGB_C4170W	-4,6	ADV21004	Conserved hypothetical protein	E6R3D2	Uncharacterized protein
		CGB_C4320C	-7,050	ADV21037	Uncharacterized protein	E6R3F0	Uncharacterized protein
GO:0007264	Small GTPase mediated signal transduction	CGB_H0200C	-6,259	ADV23784	Rho small monomeric GTPase	E6RA76	Rho small monomeric GTPase
		CGB_L0550C	-20,875	ADV25133	Uncharacterized protein	E6REL5	Uncharacterized protein
GO:0006168	Adenine salvage	CGB_I2050W	-8,222	ADV24326	Adenine phosphoribosyltransferase	E6RC06	Adenine phosphoribosyltransferase
GO:0006627	Protein processing involved in protein targeting to mitochondrion	CGB_C5520C	-4,152	ADV21102	Mitochondrial intermediate peptidase, mitochondrial precursor	E6R3N0	Mitochondrial intermediate peptidase, mitochondrial precursor
GO:0051301	Cell division	CGB_H0200C	-6,259	ADV23784	Rho small monomeric GTPase	E6RA76	Rho small monomeric GTPase
GO:0000122	Negative regulation of transcription by RNA polymerase II	CGB_A3050C	-4,608	ADV19591	Hypothetical protein	E6QZJ7	Uncharacterized protein
GO:0009435	NAD biosynthetic process	CGB_F6340C	-2,977	ADV23200	Nicotinate-nucleotide adenylyltransferase	E6R8R8	Nicotinate-nucleotide adenylyltransferase
GO:0007017	Microtubule-based process	CGB_F4090W	-4,692	ADV23017	Uncharacterized protein	E6R8A0	Dynein light chain
GO:0044804	Autophagy of nucleus	CGB_C4170W	-4,6	ADV21004	Conserved hypothetical protein	E6R3D2	Uncharacterized protein
GO:0032258	Protein localization by the Cvt pathway	CGB_C4170W	-4,6	ADV21004	Conserved hypothetical protein	E6R3D2	Uncharacterized protein
GO:1902660	Negative regulation of glucose mediated signaling pathway	CGB_A4040C	-13,322	ADV19675	Conserved hypothetical protein	E6QXL3	Serine/threonine-protein phosphatase
GO:0072462	Signal transduction involved in meiotic recombination checkpoint	CGB_A4040C	-13,322	ADV19675	Conserved hypothetical protein	E6QXL3	Serine/threonine-protein phosphatase
GO:0000447	Endonucleolytic cleavage in ITS1 to separate SSU-rRNA from 5.8S rRNA and LSU-rRNA from tricistronic rRNA transcript	CGB_D7200C	-5,346	ADV22061	Ribosomal protein s21	E6R4J4	40S ribosomal protein S21
GO:0016560	Protein import into peroxisome matrix, docking	CGB_C5400W	-4,310	ADV21071	Peroxisomal membrane protein pex13 (peroxin-13)	E6R3L8	Peroxisomal membrane protein pex13 (peroxin-13)
GO:0006105	Succinate metabolic process	CGB_A2050W	-4.555	ADV19457	Carbon utilization by utilization of organic compounds-related protein	E6QZC1	Carbon utilization by utilization of organic compounds-related protein
GO:0031098	Stress-activated protein kinase signaling cascade	CGB_I2500W	-3,829	ADV24349	Map kinase kinase kinase mkh1	E6RC52	Map kinase kinase kinase mkh1

GO:0006995	Cellular response to nitrogen starvation	CGB_C4170W	-4,6	ADV21004	Conserved hypothetical protein	E6R3D2	Uncharacterized protein
GO:0030488	tRNA methylation	CGB_G1490C	-14.902	ADV23341	Cytoplasm protein	E6R946	Cytoplasm protein
GO:0042981	Regulation of apoptotic process	CGB_I2500W	-3,829	ADV24349	Map kinase kinase kinase mkh1	E6RC52	Map kinase kinase kinase mkh1
GO:0006675	Mannosyl-inositol phosphorylceramide metabolic process	CGB_K3610C	-8,225	ADV24994	Inositolphosphorylceramide-B C-26 hydroxylase (IPC-B hydroxylase)	E6RDV9	Inositolphosphorylceramide-B C-26 hydroxylase (IPC-B hydroxylase)
GO:0006808	Regulation of nitrogen utilization	CGB_A3050C	-4,608	ADV19591	Hypothetical protein	E6QZJ7	Uncharacterized protein
GO:0009116	Nucleoside metabolic process	CGB_I2050W	-8,222	ADV24326	Adenine phosphoribosyltransferase	E6RC06	Adenine phosphoribosyltransferase
GO:0042273	Ribosomal large subunit biogenesis	CGB_I3170W	-5,389	ADV24405	Ribosomal protein	E6RC93	Ribosomal protein
GO:0000724	Double-strand break repair via homologous recombination	CGB_A4040C	-13,322	ADV19675	Conserved hypothetical protein	E6QXL3	Serine/threonine-protein phosphatase
GO:0000461	Endonucleolytic cleavage to generate mature 3'-end of SSU-rRNA	CGB_D7200C	-5,346	ADV22061	Ribosomal protein s21	E6R4J4	40S ribosomal protein S21
GO:0032147	Ativation of protein kinase activity	CGB_I2500W	-3,829	ADV24349	Map kinase kinase kinase mkh1	E6RC52	Map kinase kinase kinase mkh1
GO:0008608	Attachment of spindle microtubules to kinetochore	CGB_A3110C	-9,102	ADV19587	Hypothetical protein	E6QZK3	Uncharacterized protein
GO:0042026	Protein refolding	CGB_F6170W	-4,212	ADV23161	Hypothetical Protein	E6R8Q1	Uncharacterized protein
GO:0030154	Cell differentiation	CGB_G4250W	-8,119	ADV23507	Hypothetical protein	E6R9M3	Uncharacterized protein
GO:0006869	Lipid transport	CGB_E5610C	-5,345	ADV22646	Phosphatidylinositol transporter	E6R617	Phosphatidylinositol transporter
GO:0000045	Autophagosome assembly	CGB_C4170W	-4,6	ADV21004	Conserved hypothetical protein	E6R3D2	Uncharacterized protein
GO:0045324	Late endosome to vacuole transport	CGB_C4170W	-4,6	ADV21004	Conserved hypothetical protein	E6R3D2	Uncharacterized protein
GO:0061414	Positive regulation of transcription from RNA polymerase II promoter by a nonfermentable carbon source	CGB_N3160W	-25.174	ADV25811	Hypothetical Protein	E6RFL5	Uncharacterized protein
GO:2000002	Negative regulation of DNA damage checkpoint	CGB_A4040C	-13,322	ADV19675	Conserved hypothetical protein	E6QXL3	Serine/threonine-protein phosphatase
GO:0007346	Regulation of mitotic cell cycle	CGB_I2500W	-3,829	ADV24349	Map kinase kinase kinase mkh1	E6RC52	Map kinase kinase kinase mkh1
GO:0019441	Tryptophan catabolic process to kynurenine	CGB_C7140C	-3,758	ADV21269	Tryptophan 2,3-dioxygenase	E6R1W9	Tryptophan 2,3-dioxygenase
GO:0034553	Mitochondrial respiratory chain complex II assembly	CGB_A2050W	-4.555	ADV19457	Carbon utilization by utilization of organic compounds-related protein	E6QZC1	Carbon utilization by utilization of organic compounds-related protein
GO:0006085	Acetyl-CoA biosynthetic process	CGB_N2330W	-6.061	ADV25757	Acetate kinase	E6RG60	Probable acetate kinase
GO:0006479	Protein methylation	CGB_A3090W	-13.206	ADV19532	Conserved hypothetical protein	E6QZK1	Uncharacterized protein
GO:0045013	Carbon catabolite repression of transcription	CGB_D7530W	-7,929	ADV22011	WD-repeat protein	E6R4M6	WD-repeat protein

GO:0015976	Carbon utilization	CGB_A2050W	-4.555	ADV19457	Carbon utilization by utilization of organic compounds-related protein	E6QZC1	Carbon utilization by utilization of organic compounds-related protein
GO:2001034	Cositive regulation of double-strand break repair via nonhomologous end joining	CGB_A4040C	-13,322	ADV19675	Conserved hypothetical protein	E6QXL3	Serine/threonine-protein phosphatase
GO:0006099	Tricarboxylic acid cycle	CGB_A2040C	-6,668	ADV19527	Isocitrate dehydrogenase (NADP+)	E6QZC0	Isocitrate dehydrogenase [NADP]
GO:0070158	Mitochondrial seryl-tRNA aminoacylation	CGB_B9350W	-3,084	ADV20640	Seryl-tRNA synthetase	E6R1G0	Seryl-tRNA synthetase
GO:0035023	Regulation of Rho protein signal transduction	CGB_L0550C	-20,875	ADV25133	Uncharacterized protein	E6REL5	Uncharacterized protein
GO:0000398	mRNA splicing, via spliceosome	CGB_F1330C	-4,158	ADV22872	Formin binding protein 3	E6R7S2	Formin binding protein 3
GO:0006749	Glutathione metabolic process	CGB_J1670W	-6,9	ADV24625	Glutathione transferase	E6RCV4	Glutathione transferase
GO:0006779	Porphyrin-containing compound biosynthetic process	CGB_E1210C	-4,233	ADV22386	Coproporphyrinogen oxidase	E6R6J6	Coproporphyrinogen oxidase
GO:0002943	tRNA dihydrouridine synthesis	CGB_B2580W	-3,454	ADV20241	Conserved hypothetical protein	E6R0A8	Uncharacterized protein
GO:0006111	Regulation of gluconeogenesis	CGB_A2050W	-4.555	ADV19457	Carbon utilization by utilization of organic compounds-related protein	E6QZC1	Carbon utilization by utilization of organic compounds-related protein
GO:0034755	Iron ion transmembrane transport	CGB_B5580W	-4,090	ADV20475	Iron ion transporter	E6R106	Iron ion transporter
GO:0000209	Protein polyubiquitination	CGB_A3660C	-10,45	ADV20032	RING zinc finger protein	E6QXK9	RING zinc finger protein
GO:0006102	Isocitrate metabolic process	CGB_A2040C	-6,668	ADV19527	Isocitrate dehydrogenase (NADP+)	E6QZC0	Isocitrate dehydrogenase [NADP]
GO:0043161	Proteasome-mediated ubiquitin-dependent protein catabolic process	CGB_A3660C	-10,45	ADV20032	RING zinc finger protein	E6QXK9	RING zinc finger protein
GO:0031930	Mitochondria-nucleus signaling pathway	CGB_A3050C	-4,608	ADV19591	Hypothetical protein	E6QZJ7	Uncharacterized protein
GO:0006470	Protein dephosphorylation	CGB_A4040C	-13,322	ADV19675	Conserved hypothetical protein	E6QXL3	Serine/threonine-protein phosphatase
GO:0046416	D-amino acid metabolic process	CGB_E0280W	-8,087	ADV22267	D-amino-acid oxidase	E6R6D0	D-amino-acid oxidase
GO:0097502	Mannosylation	CGB_H1610W	-5,650	ADV23826	Alpha-1,6-mannosyltransferase	E6RAJ5	Alpha-1,6-mannosyltransferase
GO:0016579	Protein deubiquitination	CGB_D7530W	-7,929	ADV22011	WD-repeat protein	E6R4M6	WD-repeat protein
GO:0023014	Signal transduction by protein phosphorylation	CGB_I2500W	-3,829	ADV24349	Map kinase kinase kinase mkh1	E6RC52	Map kinase kinase kinase mkh1
GO:0046323	Glucose import	CGB_B1090C	-3,715	ADV20206	ITR1	E6QZX9	ITR1
GO:0000413	Protein peptidyl-prolyl isomerization	CGB_F6170W	-4,212	ADV23161	Hypothetical Protein	E6R8Q1	Uncharacterized protein
	No GO Terms	CGB_A1160C	-9,6	ADV19450	Hypothetical Protein	E6QZ47	Uncharacterized protein
		CGB_A1070C	-3,622	ADV19454	Negative regulator of differentiation 1 (Multicopy suppressor of sporulation protein msa2)	E6QZ38	Negative regulator of differentiation 1 (Multicopy suppressor of sporulation protein msa2)
		CGB_A1170W	-9,582	ADV19384	Guanosine-diphosphatase	E6QZ48	Guanosine-diphosphatase
		CGB_A4030C	-5,787	ADV19676	Glyoxal oxidase precursor	E6QXL2	Glyoxal oxidase
		CGB_A4370W	-3,301	ADV19608	Cyclin	E6QXP6	Cyclin
		CGB_A4450W	-5,316	ADV19612	3-isopropylmalate dehydrogenase	E6QXQ4	3-isopropylmalate dehydrogenase

		CGB_A4510W	-3,772	ADV19617	Glutamate-ammonia ligase	E6QXR0	Glutamine synthetase
		CGB_A5490C	-8,406	ADV19734	Membrane transport protein	E6QXZ3	Membrane transport protein
		CGB_A8570W	-8,035	ADV19917	MAP kinase kinase	E6QYM2	MAP kinase kinase
		CGB_A9140C	-8,745	ADV20012	Multidrug resistance protein 1	E6QYP8	Multidrug resistance protein 1
		CGB_A9330C	-6,036	ADV20001	Microtubule binding protein	E6QYR7	Autophagy-related protein
		CGB_B0060C	-2,944	ADV20128	Methionine-tRNA ligase	E6QZP2	Methionine-tRNA ligase
		CGB_B1130W	-7,022	ADV20139	Hsp90 cochaperone	E6QZY3	Hsp90 cochaperone
		CGB_B1200C	-4,776	ADV20200	Two-component sensor molecule,	E6QZZ0	Two-component sensor molecule,
		CGB_B3630C	-5,099	ADV20353	Alpha/beta hydrolase protein	E6R0K5	Alpha/beta hydrolase protein
		CGB_B4440W	-7,312	ADV20403	Golgi to plasma membrane transport-related protein	E6R0S3	Golgi to plasma membrane transport-related protein
		CGB_B6250C	-8,264	ADV20590	CAP64 gene product - related protein	E6R156	CAP64 gene product - related protein
		CGB_B6560C	-3,689	ADV20573	mRNA guanylyltransferase	E6R187	mRNA-capping enzyme subunit alpha
		CGB_C0030C	-3,081	ADV20782	Flavohemoprotein (Hemoglobin-like protein)	E6R2I3	Flavohemoprotein (Hemoglobin-like protein)
		CGB_C2120W	-8,214	ADV20861	Polyamine transport-related protein	E6R2Y2	Polyamine transport-related protein
		CGB_C3150C	-4,9791	ADV20987	Histone deacetylase 1-1 (hd1)	E6R363	Histone deacetylase
		CGB_C7540W	-4,14	ADV21231	Protein serine/threonine phosphatase 4 regulatory subunit 1	E6R209	Protein serine/threonine phosphatase 4 regulatory subunit 1
		CGB_C8520W	-4,134	ADV21301	Phosphoribosyl-ATP diphosphatase	E6R282	Phosphoribosyl-ATP diphosphatase
		CGB_C8770C	-6,479	ADV21443	Glycerate-and formate-dehydrogenase	E6R2A7	Glycerate-and formate-dehydrogenase
		CGB_C9060C	-5,391	ADV21417	Chaperone regulator	E6R2B4	Chaperone regulator
		CGB_D3660C	-6,666	ADV21719	Cytoplasm protein	E6R3R7	Cytoplasm protein
		CGB_D4470W	-3,703	ADV21770	Aldehyde dehydrogenase (ALDDH)	E6R3X5	Aldehyde dehydrogenase (ALDDH)
		CGB_D5300C	-4,410	ADV21904	Delta-12 fatty acid desaturase	E6R445	Delta-12 fatty acid desaturase
		CGB_D6180C	-3,816	ADV21980	Cytochrome b2, mitochondrial precursor (L-lactate ferricytochrome C oxidoreductase)	E6R4C0	Cytochrome b2, mitochondrial precursor (L-lactate ferricytochrome C oxidoreductase)
		CGB_D6200C	-3,866	ADV21978	Arginase	E6R4C2	Arginase
		CGB_D8210W	-3,240	ADV22083	Carboxylic acid transport protein	E6R4T2	Carboxylic acid transport protein
		CGB_D8500C	-7,076	ADV22125	Ada3 protein (Ngg1 protein)	E6R4W1	Ada3 protein (Ngg1 protein)

		CGB_D8620C	-5,087	ADV22119	Pyruvate decarboxylase	E6R4X3	Pyruvate decarboxylase
		CGB_D8660W	-15,976	ADV22105	Rab family GTP-binding protein	E6R4X7	Rab family GTP-binding protein
		CGB_D9130W	-4,048	ADV22155	Adaptation to pheromone during conjugation with cellular fusion-related protein	E6R503	Adaptation to pheromone during conjugation with cellular fusion-related protein
		CGB_D9180C	-6,345	ADV22219	Response to drug-related protein	E6R508	Response to drug-related protein
		CGB_E1220W	-3,644	ADV22336	Ubiquinone biosynthesis-related protein	E6R6J7	Ubiquinone biosynthesis-related protein
		CGB_E3690C	-4,483	ADV22507	Vacuolar import and degradation protein	E6R746	Vacuolar import and degradation protein
		CGB_E5410W	-7,148	ADV22624	Methionyl-tRNA formyltransferase	E6R5Z7	Methionyl-tRNA formyltransferase
		CGB_F3680C	-3,784	ADV22980	Regulation of carbohydrate metabolism-related protein	E6R887	Regulation of carbohydrate metabolism-related protein
		CGB_F5010W	-7,112	ADV23082	Transcription-related protein	E6R8G2	Transcription-related protein
		CGB_F6320W	-4,128	ADV23169	Nucleoside diphosphatase	E6R8R6	Nucleoside diphosphatase
		CGB_H0580C	-68	ADV23766	Nicotinamide mononucleotide permease	E6RAB4	Nicotinamide mononucleotide permease
		CGB_H0590W	-281,857	ADV23747	Tartarate dehydrogenase	E6RAB5	Tartarate dehydrogenase
		CGB_H0710C	-8,675	ADV23747	Glyceraldehyde 3-phosphate dehydrogenase	E6RAC7	Glyceraldehyde 3-phosphate dehydrogenase
		CGB_H3560C	-8,211	ADV23996	Vacuolar protein sorting 41, putative	E6RAZ5	Vacuolar protein sorting-associated protein 41
		CGB_I4200W	-3,843	ADV24474	Histone deacetylase 1 (hd1)	E6RCG5	Histone deacetylase
		CGB_I4220C	-8,364	ADV24518	Ubiquitin-protein ligase	E6RCG7	Ubiquitin-protein ligase
		CGB_I4610C	-6,573	ADV24492	High-affinity glucose transporter of the major facilitator superfamily; Hxt2p	E6RBL5	High-affinity glucose transporter of the major facilitator superfamily; Hxt2p
		CGB_J0050C	-4,203	ADV24597	Cytoplasm protein	E6RCH9	Cytoplasm protein
		CGB_J1070W	-5,708	ADV24601	Telomere maintenance protein	E6RCP4	Telomere maintenance protein
		CGB_J1280W	-27,449	ADV24608	Stomatin-like protein	E6RCR5	Stomatin-like protein
		CGB_K2180W	-10,097	ADV24890	Actin-like protein ARP5	E6RDJ3	Actin-like protein ARP5
		CGB_K4160W	-4,625	ADV25031	Autophagy-related protein	E6RDY8	Autophagy-related protein
		CGB_L3200W	-3,468	ADV25316	Cysteine synthase	E6REA3	Cysteine synthase
		CGB_M0350C	-28,931	ADV25429	Aryl-alcohol dehydrogenase	E6RF10	Aryl-alcohol dehydrogenase
		CGB_M3480C	-13,594	ADV25598	Iron transport multicopper oxidase FET3 precursor	E6RFK1	Iron transport multicopper oxidase FET3
		CGB_M3490W	-7,561	ADV25588	Iron transporter	E6RFK2	Iron transporter

		CGB_N3350C	-19,365	ADV25841	1,3-beta-glucanosyltransferase	E6RFN4	1,3-beta-glucanosyltransferase
		CGB_A4100W	-7,421	ADV19596	Hypothetical protein	E6QXL9	Uncharacterized protein
		CGB_A4700W	-4,390	ADV19628	Hypothetical protein	E6QXS9	Uncharacterized protein
		CGB_A4860C	-10,658	ADV20035	Hypothetical protein	E6QXU5	Uncharacterized protein
		CGB_A7500C	-9,307	ADV19867	Hypothetical protein	E6QYE4	Uncharacterized protein
		CGB_B0390C	-5,146	ADV20111	Hypothetical protein	E6QZS7	Uncharacterized protein
		CGB_B1590W	-3,166	ADV20162	Conserved hypothetical protein	E6R028	Protein transport protein sec16
		CGB_B1670C	-4,084	ADV20179	Conserved hypothetical protein	E6R036	Uncharacterized protein
		CGB_B3380W	-4,482	ADV20317	Hypothetical protein	E6R0I0	Uncharacterized protein
		CGB_B4150C	-4,256	ADV20442	Hypothetical Protein	E6R0P4	Uncharacterized protein
		CGB_B4160C	-3,489	ADV20441	Hypothetical Protein	E6R0P5	Uncharacterized protein
		CGB_B5210C	-5,817	ADV20520	Hypothetical protein	E6R0W8	Uncharacterized protein
		CGB_B6590W	-5,566	ADV20556	Hypothetical protein	E6R190	Uncharacterized protein
		CGB_B9370C	-6,183	ADV20682	Hypothetical protein	E6R1G2	Uncharacterized protein
		CGB_C0070C	-5,988	ADV20778	Hypothetical Protein	E6R2I7	Uncharacterized protein
		CGB_C0240W	-6	ADV20723	Hypothetical Protein	E6R2K4	Uncharacterized protein
		CGB_C0680C	-5,903	ADV20751	Conserved hypothetical protein	E6R2P7	Uncharacterized protein
		CGB_C1190C	-6,845	ADV20847	Uncharacterized protein	E6R2R9	Uncharacterized protein
		CGB_C2130C	-4,945	ADV20924	Uncharacterized protein	E6R2Y3	Uncharacterized protein
		CGB_C2150W	-3,406	ADV20863	Hypothetical Protein	E6R2Y5	Uncharacterized protein
		CGB_C3370C	-3,819	ADV20975	Hypothetical Protein	E6R385	Uncharacterized protein
		CGB_C4330W	-3,115	ADV21012	Hypothetical Protein	E6R3F1	Uncharacterized protein
		CGB_C4390W	-10,693	ADV21015	Hypothetical Protein	E6R3F7	Uncharacterized protein
		CGB_C5420C	-3,356	ADV21105	Uncharacterized protein	E6R3M0	Uncharacterized protein
		CGB_C6620C	-3,405	ADV21171	Conserved hypothetical protein	E6R1T9	Uncharacterized protein
		CGB_C8010W	-4,242	ADV21274	Uncharacterized protein	E6R231	Uncharacterized protein
		CGB_C8200W	-3,142	ADV21284	Hypothetical Protein	E6R250	Uncharacterized protein
		CGB_C8410W	-3,559	ADV21295	Hypothetical Protein	E6R271	Uncharacterized protein
		CGB_C9340C	-8,809	ADV21402	Uncharacterized protein	E6R2E2	Uncharacterized protein
		CGB_D4180C	-3,675	ADV21826	Hypothetical Protein	E6R3U6	Uncharacterized protein
		CGB_D4230W	-4,777	ADV21762	Hypothetical protein	E6R3V1	Uncharacterized protein
		CGB_D6660W	-6,018	ADV21954	Hypothetical protein	E6R4G9	Uncharacterized protein
		CGB_D8630W	-11,83	ADV22103	Hypothetical protein	E6R4X4	Uncharacterized protein
		CGB_D9110C	-4,470	ADV22223	Hypothetical protein	E6R501	Uncharacterized protein
		CGB_D9510C	-6,515	ADV22201	Hypothetical protein	E6R541	Uncharacterized protein
		CGB_E0320W	-6,638	ADV22270	Hypothetical protein	E6R6D4	Uncharacterized protein
		CGB_E1280C	-10,551	ADV22384	Uncharacterized protein	E6R6K3	Uncharacterized protein
		CGB_E1550C	-3,576	ADV22372	Hypothetical Protein	E6R6N0	Uncharacterized protein

		CGB_E5100W	-22,545	ADV22609	Hypothetical Protein	E6R7C7	Uncharacterized protein
		CGB_E5400C	-3,915	ADV22655	Conserved hypothetical protein	E6R5Z6	Uncharacterized protein
		CGB_E6470W	-4,351	ADV22694	Hypothetical protein	E6R674	Uncharacterized protein
		CGB_F0310W	-3,176	ADV22777	Conserved hypothetical protein	E6R7L2	Uncharacterized protein
		CGB_F1590W	-4,677	ADV22848	Uncharacterized protein	E6R7U8	Rhomboid-like protein
		CGB_F4130C	-5,632	ADV23073	Conserved hypothetical protein	E6R8A4	Uncharacterized protein
		CGB_F4310C	-16,125	ADV23065	Uncharacterized protein	E6R8C2	Uncharacterized protein
		CGB_F5150C	-8,8	ADV23146	Hypothetical Protein	E6R8H6	Uncharacterized protein
		CGB_F6190W	-5,276	ADV23162	Hypothetical protein	E6R8Q3	Uncharacterized protein
		CGB_F6355C	-25,264	ADV23198	Hypothetical protein	E6R7F1	Uncharacterized protein
		CGB_G1580C	-4,204	ADV23337	Hypothetical protein	E6R955	Uncharacterized protein
		CGB_G1670C	-10,460	ADV23706	Hypothetical protein	E6R964	Protein yippee-like
		CGB_G3170C	-9,489	ADV23486	Conserved hypothetical protein	E6R9E3	Uncharacterized protein
		CGB_G5140W	-7,619	ADV23576	Hypothetical Protein	E6R9T9	Uncharacterized protein
		CGB_H0600W	-15,122	ADV23748	Hypothetical protein	E6RAB6	Uncharacterized protein
		CGB_H1550W	-6,137	ADV23822	Hypothetical Protein	E6RAI9	Uncharacterized protein
		CGB_H3260C	-8,625	ADV24013	Conserved hypothetical protein	E6RAW4	Uncharacterized protein
		CGB_H5430C	-13,806	ADV24160	Uncharacterized protein	E6RBE3	Uncharacterized protein
		CGB_H5750W	-9,118	ADV24138	Hypothetical protein	E6RBH5	Uncharacterized protein
		CGB_I2195W	-4,666	ADV24362	Hypothetical Protein	E6RC21	Uncharacterized protein
		CGB_I2310W	-4,363	ADV24340	Hypothetical Protein	E6RC33	Uncharacterized protein
		CGB_I3340W	-4,620	ADV24414	Hypothetical Protein	E6RCB0	Uncharacterized protein
		CGB_I3480W	-75,909	ADV24420	Conserved hypothetical protein	E6RCC4	Uncharacterized protein
		CGB_I4530C	-4,528	ADV24496	Conserved hypothetical protein	E6RBK7	Uncharacterized protein
		CGB_J0130W	-18,410	ADV24545	Hypothetical protein	E6RCI9	Uncharacterized protein
		CGB_J0440W	-9,190	ADV24564	Hypothetical protein	E6RCL9	Uncharacterized protein
		CGB_J0570C	-9,005	ADV24574	Hypothetical protein	E6RCN3	Uncharacterized protein
		CGB_J1050W	-5,008	ADV24600	Hypothetical protein	E6RCP2	Uncharacterized protein
		CGB_J1060C	-12,326	ADV24662	Hypothetical protein	E6RCP3	Uncharacterized protein
		CGB_J1590W	-5,785	ADV24622	Hypothetical protein	E6RCU6	Uncharacterized protein
		CGB_J2120C	-5,604	ADV24712	Uncharacterized protein	E6RCW7	Uncharacterized protein
		CGB_J2260C	-5,9	ADV24705	Conserved hypothetical protein	E6RCY1	Uncharacterized protein
		CGB_J2280C	-4,434	ADV24704	Hypothetical protein	E6RCY3	Uncharacterized protein
		CGB_K1750C	-7,696	ADV24848	Conserved hypothetical protein	E6RDH0	Uncharacterized protein

		CGB_K2150C	-3,818	ADV24945	Conserved hypothetical protein	E6RDJ0	Uncharacterized protein
		CGB_K3140W	-12,2	ADV24960	Hypothetical protein	E6RDR2	Uncharacterized protein
		CGB_K3290W	-5,529	ADV24967	Conserved hypothetical protein	E6RDS7	Uncharacterized protein
		CGB_K3620C	-6,8	ADV24993	Hypothetical protein	E6RDW0	Uncharacterized protein
		CGB_K4360C	-3,797	ADV25070	Hypothetical protein	E6RE08	Uncharacterized protein
		CGB_L0540C	-7,796	ADV25134	Uncharacterized protein	E6REL4	Uncharacterized protein
		CGB_L1190W	-11,879	ADV25172	Hypothetical protein	E6REP5	Uncharacterized protein
		CGB_L1690W	-8,272	ADV25386	Hypothetical protein	E6REU7	Uncharacterized protein
		CGB_L2110C	-38,271	ADV25301	Uncharacterized protein	E6REV6	Uncharacterized protein
		CGB_L2220W	-6,35	ADV25240	Conserved hypothetical protein	E6REW7	Uncharacterized protein
		CGB_L2510W	-5,504	ADV25254	Hypothetical protein	E6RE56	Uncharacterized protein
		CGB_M1320C	-9,482	ADV25489	Hypothetical protein	E6RF66	Uncharacterized protein
		CGB_M3170W	-11,254	ADV25570	Hypothetical protein	E6RFH0	Uncharacterized protein
		CGB_M3500W	-9,503	ADV25589	Hypothetical protein	E6RFK3	Uncharacterized protein
		CGB_N0250W	-6,22	ADV25636	Hypothetical protein	E6RFT2	Uncharacterized protein
		CGB_N1550C	-6,066	ADV25711	Hypothetical protein	E6RG15	Uncharacterized protein
